# Individual Differences in Indirect Speech Act Processing Found Outside the Language Network

**DOI:** 10.1162/nol_a_00066

**Published:** 2022-04-13

**Authors:** Katarina Bendtz, Sarah Ericsson, Josephine Schneider, Julia Borg, Jana Bašnáková, Julia Uddén

**Affiliations:** Department of Psychology, Stockholm University, Sweden; Donders Centre for Cognitive Neuroimaging, Nijmegen, The Netherlands; Institute of Experimental Psychology, Centre of Social and Psychological Sciences SAS, Slovakia; Department of Linguistics, Stockholm University, Sweden

**Keywords:** pragmatics, communication, fMRI, Indirect speech acts, language, theory of mind

## Abstract

Face-to-face communication requires skills that go beyond core language abilities. In dialogue, we routinely make inferences beyond the literal meaning of utterances and distinguish between different speech acts based on, e.g., contextual cues. It is, however, not known whether such communicative skills potentially overlap with core language skills or other capacities, such as theory of mind (ToM). In this functional magnetic resonance imaging (fMRI) study we investigate these questions by capitalizing on individual variation in pragmatic skills in the general population. Based on behavioral data from 199 participants, we selected participants with higher vs. lower pragmatic skills for the fMRI study (*N* = 57). In the scanner, participants listened to dialogues including a direct or an indirect target utterance. The paradigm allowed participants at the whole group level to (passively) distinguish indirect from direct speech acts, as evidenced by a robust activity difference between these speech acts in an extended language network including ToM areas. Individual differences in pragmatic skills modulated activation in two additional regions outside the core language regions (one cluster in the left lateral parietal cortex and intraparietal sulcus and one in the precuneus). The behavioral results indicate segregation of pragmatic skill from core language and ToM. In conclusion, contextualized and multimodal communication requires a set of interrelated pragmatic processes that are neurocognitively segregated: (1) from core language and (2) partly from ToM.

## INTRODUCTION

Dialogue constitutes a fundamental form of human communication (Levinson, 2006). In accordance with this suggestion, being able to communicate face-to-face with another person may require skills that go beyond “core” language skills, such as lexical retrieval or syntactic composition. Communication in real-world settings places specific requirements on the interlocutors, for example, the ability to go beyond the literal meaning of utterances based on contextual cues to understand the speech acts forming the basis of conversational turns, or to tailor each utterance to the perspective or knowledge of the addressee. There is an ongoing debate on the nature of these so-called *pragmatic* skills and the extent to which they overlap with different core language skills or other capacities, such as executive functions or theory of mind (ToM) skills (see, e.g., Matthews et al., 2018, for a developmental approach). Behavioral investigations of conversational implicature comprehension suggest that the pragmatic skills necessary for interpreting at least some aspects of dialogue are relatively independent of structural language skills, e.g., vocabulary and syntax (Wilson & Bishop, 2019).

### Neurocognitive Dissociation of Pragmatics from Core Language, ToM, and Cognitive Control Functions

In this article, we adopt a neuroscience approach to contribute an additional perspective on the complex knowledge domain of pragmatics and its representation in the human brain. This approach enables us to investigate whether there are specific neural signatures of pragmatic skills, or whether these skills draw on general cognitive skills, as would be indicated by modulation of activity in three other networks: the left-lateralized perisylvian language network, the ToM network, and the multiple demand (MD) network subserving executive functions. As our primary test bed, we chose indirect speech acts (ISAs), since interpreting their communicative meaning requires fully fledged context-based pragmatic inferencing.

Our approach capitalizes on the fact that even within the neurotypical population, there is substantial variability in pragmatic skills. By combining neuroimaging techniques with behavioral measurements of this variability, we investigate several novel questions. First, we ask whether, where, and how individual pragmatic variability will manifest itself in neural activity during pragmatic inferencing. More specifically, we wonder where this variability will be manifested in relation to the three networks specified above. Second, we ask to what extent individual behavioral and neural pragmatic variability can be explained away by variability in core language skills, ToM skills, and executive functions. If it cannot, this would be a sign of neurocognitive segregation of pragmatics from the other aspects of cognition. In other words, such a segregation would mean that neither core language processes, nor core language processes together with ToM, nor these two domains together with cognitive control processes suffice to fully characterize pragmatic processing. A theoretical reason that we study these three cognitive skills in relation to pragmatics is that they can be easily argued to influence or be influenced by pragmatics during the development of language and communication. Indeed, these three skills have received most attention in relation to pragmatic skill in developmental research (Matthews et al., 2018).

### The Indirect Speech Acts fMRI Experiment – Dissociations from Core Language

Interpreting the communicative meaning of ISAs requires pragmatic inferences about what the speakers truly meant. Thus, in order to measure individual differences in pragmatic processing in the functional magnetic resonance imaging (fMRI) scanner, we used an established neurocognitive paradigm on ISA processing. In this paradigm, called the *indirect speech acts experiment*, participants listened to a dialogue including an introductory context and a target utterance with literal and prosodic cues. In several previous studies, the paradigm has allowed most participants to (passively) discern indirect from direct speech acts, as evidenced by robust activity differences between these two speech acts in the core language network, but crucially also in an extended language network including ToM/mentalizing areas in young adults (Bašnáková et al., 2014; van Ackeren et al., 2016) as well as adolescents (Asaridou et al., 2019). The effect has been stable across written and auditory versions of the experiment and is also present when gestures provide the target information expressing the direct or indirect speech act (Bašnáková, 2019). Two studies have in addition found areas beyond core language areas, including mentalizing areas, in related pragmatic contrasts: Egorova et al. (2016) contrasted two different direct speech acts, and Hellbernd and Sammler (2016, 2018), where participants categorized speech acts based on prosody.These existing ISA results thus already provide neural evidence that pragmatic processes cannot be reduced to core language processes. Moreover, if we compare areas reported in these studies to the neurosynth mask (i.e., a meta-analytical statistical activation map associated with a keyword) based on the term *language comprehension*, we see overlap with the ISA literature in both medial frontal and the bilateral temporoparietal junction (TPJ) regions, which are also not considered part of the core language network. In addition, the typical left-hemisphere dominance of the perisylvian language network is absent, in favor of a more bilaterally balanced (Asaridou et al., 2019; Egorova et al., 2016; Hellbrend & Sammler, 2018) or a right dominant activation pattern (Bašnáková, 2019). We expected not only that we would replicate the previously found areas but also that individual variability across groups would be manifested in the vicinity (localization-wise) of these areas, although the exact location was more difficult to predict, as the study is the first investigating *individual variability* in higher-level pragmatic inferencing.

We use the term *language network* to refer to the cortical areas recruited for several interrelated processes that at the same time are partly separable from a neurocognitive point of view. Depending on the perspective taken, core language processing can, for instance, be considered as separable lexical, combinatorial, phonological, syntactic, and semantic processes, as well as comprehension and production processes (Hagoort, 2017). The included areas (inferior frontal, anterior temporal, and posterior temporal cortices, in particular from the middle temporal gyrus (MTG) and up, as well as the angular gyrus) are highly characteristic, often observed bilaterally but with left dominance (although lateralization depends on the process studied). The network is to some extent modular and distinguishable as a resting state network (Paunov et al., 2019), yet at the same time it is possible to divide it into, for example, dorsal vs. ventral pathways (Friederici, 2012; Hickok & Poeppel, 2004).

It should also be noted that some areas in the language network (e.g., the inferior frontal gyrus (IFG) and the posterior superior temporal cortices) are commonly observed in other tasks, possibly also sometimes in ToM related tasks (Schurz et al., 2014, 2015). Using factor analysis Wilson and Bishop (2019) showed that a two-factor model, using pragmatic skill and core language skill as two separate factors, captures variation in young healthy adults better than a one-factor model, although the two factors are robustly related. However, in a large-scale meta-analysis across lifespan, figurative language use has been shown to be highly related to core language skills in autism spectrum disorders (ASD; Kalandadze et al., 2018). We wanted to test the extent to which the pragmatic skills and processes we measured depended on individual differences in, for example, reading speed, vocabulary, word recognition, speed of lexical access, or cultural literacy (see a description of used tasks in the section: Behavioral tests of language ability). Accordingly, participants were given a series of tests measuring their formal language abilities: nonpragmatic or core linguistic skills as measured by tests for print exposure/reading skill (the author recognition test (AR); Moore & Gordon, 2015), lexical access (the lexical decision task (LDT); Holmer et al., 2016), and vocabulary (from the Vocabulary, Swedish Scholastic Aptitude Test (vSweSAT); Cliffordson, 2004); see the section: Behavioral tests of language ability. Our expectation was that individual variance in core language processes would neither suffice alone to account for variance in pragmatics, nor suffice when combined with variation in ToM processes (see a description of the used ToM task in the section: Low/high pragmatic ability groups). We now turn to discuss the latter.

### No Unitary Theory of Mind Skill

Based on theoretical predictions (Grice, 1975), understanding what speakers really mean is primarily achieved through arriving at the underlying intentions behind specific utterances. Under this view, widely accepted in the field of pragmatics, communicative meaning interpretation should fall under the scope of ToM processing, as intentions are unobservable mental states (similar to beliefs and desires). However, firstly, it is now clear that ToM is not well-defined, in particular not as one domain-general function. Recent meta-analyses indicate that different categories of tasks result in different activation patterns (Schurz et al., 2014, 2015), even though there are core hubs that span across all types of tasks, for example, the hallmark task of false-belief reasoning (bilateral TPJ and medial prefrontal cortex (mPFC); Schurz et al., 2014). Along these lines, Enrici et al. (2019) suggested that communicative intention processing should be considered a fundamental cognitive component of a *reformulated* ToM system (for further details see Schaafsma et al., 2015). Secondly, many pragmatic phenomena may not require access to the mental state of the speaker in the first place—see, for example, *I’ve eaten breakfast* meaning “I’ve eaten breakfast today” (Garrett & Harnish, 2007) and *It’s cold in here* as an indirect request (Kissine, 2016)—suggesting the existence of pragmatic processes distinguished from mentalizing. Thirdly, we have reasons to question evidence from developmental studies and clinical populations showing covariation between ToM and pragmatic skills (Domanesci & Bambini, 2020). As Bosco and colleagues (2018) argue, the extent of the correlations between pragmatic phenomena and ToM is probably inflated because tests used to assess ToM are often, in fact, pragmatic tasks; for example, the strange stories test used to assess advanced ToM reasoning instead probes the implicit and explicit understanding of figurative language and irony (Bosco et al., 2018). Thus, it is generally not helpful to equate pragmatic processing skills with ToM a priori. We need empirical studies where a reduction of pragmatic processes to ToM is not assumed. Designs should instead allow for a distinction to be made between the processes. The extent to which segregation is possible should then be left as an empirical question. Including measures of core language skills in the same experiment is also crucial.

If pragmatics is indeed not simply reducible to ToM plus core language skills, this also makes clear predictions for patterns of individual variation in the general population. Behaviorally, these predictions include small or absent correlations of pragmatic skills vs. core language and ToM skills. This constitutes the basic rationale of the empirical tests in the current study. We measured 199 participants from the young adult general population using a novel behavioral battery. Based on a subset of these tests measuring two different pragmatic skills (see descriptions in the section: Behavioral tests of pragmatic ability), we selected two groups of participants with good (top 50% performers, a *high scoring* (HS) group) vs. poor (lowest 50% performers, a *low scoring* (LS) group) communicative skill for the fMRI study (*N* = 57, where 29 participants had poor communicative skills). The pragmatic skills tested for this preselection included both production and comprehension. The participants’ mentalizing (or ToM) ability was also assessed using a nonverbal test, the *reading the mind in the eyes* test (RMET; Baron-Cohen et al., 2001; see the section: Non-verbal behavioral ToM).

### Cognitive Control Functions and the MD Network

Cognitive control functions (CCFs) allow us to plan, control, and regulate complex higher-order tasks in a flexible manner. For instance, the transient representation and manipulation of task-relevant information in working memory is needed for proper cognitive control. (Note that for our purposes here, CCFs will be used interchangeably with *executive functions*.) In the current study, we ask whether proficiency in pragmatic processes is dissociable from CCFs. We start tackling this question mainly by studying individual variation in behavioral pragmatic skills in relation to CCFs, more specifically variance in a complex operation span task (OSpan; Foster et al., 2015). Inhibitory control and working memory have been shown to be associated with performance on some pragmatic tasks (Matthews et al., 2018), where for instance Ryskin et al. (2014) have used the Ospan task specifically. The OSpan task is further described in the section: Cognitive control – Operation span (OSpan).

As mentioned above, there is also a neural perspective on this matter. One of the questions in the current study is where, in terms of large-scale brain networks, individual variation in the ISA paradigm presented above will manifest itself. The three most relevant large-scale brain networks we consider are the core language network, the ToM network, and the MD network (Duncan, 2010). These networks were described in detail in a series of studies from the Fedorenko lab (Blank et al., 2014; Paunov et al., 2019), including internal replication experiments. The MD network specifically has been emerging from fMRI data as a relevant network across varied tasks (e.g., cognitive control tasks including working memory, attentional control, or general intelligence) but how to best describe its function is less clear (Duncan, 2010). A domain-general function is, however, often assumed, and hence the network is also sometimes referred to as the *task-positive network*, or the *frontoparietal control network* (FPCN; Chein et al., 2011; Vincent et al., 2008). In previous research it has been shown that the OSpan task we use indeed activates the intended MD network (Faraco et al., 2011). There have also been indications that individuals with higher OSpan performance activate this network more during the task (Faraco et al., 2011; Osaka et al., 2003). Anatomically, three reviews or meta-analyses converge on the MD network as consisting of areas in bilateral dorsolateral prefrontral cortex (e.g., BA 9/46), bilateral parietal areas (e.g., BA 7), and dorsal medial prefrontal/anterior cingulate (e.g., BA 32), and/or medial presupplementary/supplementary motor cortex (Chein et al., 2011; Duncan, 2010; Vincent et al., 2008). These studies also agree on using the label *cognitive control* (or alternatively *executive functions*, which is largely used interchangeably in the literature) when discussing the cognitive function of this network. As we thought there was a substantial probability that CCFs would influence the ISA task and since this work focuses on individual variation in communicative skills, we decided to match the LS and HS group on their CCF performance.

### Approach Summary

Our approach can be summarized with six points. (1) Two sets of experiments were conducted, an fMRI experiment and a preceding behavioral experiment. (2) We used the established ISA paradigm in the fMRI experiment to compare two conditions: indirect vs. direct speech acts. (3) We compared activity between the HS and LS groups that were formed based on the behavioral experiment of a larger sample (*N* = 199). We used two behavioral pragmatic tasks in this behavioral experiment, while at the same time controlling for what we perceived as the general cognitive skill most likely to actually influence the ISA variability, which was CCF. (4) By design, the regions showing a Group × Condition interaction are assumed to be involved in pragmatic inference. (5) To test for neurocognitive segregation, regions in (4) are compared with neurosynth networks for core language, ToM, and CCF. (6) In a further test of segregation, individual activity in regions in (4) that were further tested in (5) and do not show overlap with neurosynth networks is correlated with the following skills measured in the behavioral battery: nonpragmatic (“core”) language – vocabulary (vSweSat) and lexical access (LDT); ToM – nonverbal test of mentalizing ability (RMET); and CCF– OSpan. In addition, all behavioral tasks used are correlated with each other, testing whether pragmatic skills segregate from core language, ToM, and CCF at the cognitive level. We consider absences of overlap in (5) and nonsignificant correlations in (6) as evidence of neurocognitive segregation.

## MATERIALS AND METHODS

### Participants

Sixty participants (age 18–36, 28 males) were recorded in the fMRI experiment. No participant had any history of neurological impairment, brain surgery, or ASD/Asperger diagnosis. Participants with a history of language impairment were generally excluded prior to participation. They all gave informed consent and received 350 in SEK [Swedish krona] for their participation. The study was approved by the Swedish Regional Ethical Review Authority in Stockholm.

The participants of the fMRI experiment were selected from a larger group who participated in a preceding behavioral experiment (*N* = 199; 99 males; average age 28.7 (males) and 29.3 (females)). Participants were invited into the fMRI experiment based on their results in two tests in the behavioral experiment battery: audience design (AD) and prosodic comprehension of request for response (PC-RR; see the section: Behavioral Tests of ToM and Cognitive Control. For more information on how we formed these groups, see the section: fMRI Task: The indirect speech acts experiment.) Further details on the participants are given in the supplementary material and Table S1 (Supporting Information can be found at https://doi.org/10.1162/nol_a_00066).

### Behavioral Tests of Pragmatic Ability

#### Prosodic comprehension of requests for response

We wanted to cover a wide range of pragmatic skills, crossing the division of production and comprehension without neglecting the multimodal aspects of communicative signals. For the comprehension test, we settled on testing participants’ ability to identify a speaker’s request for feedback in a communicative situation based on prosody. Speech prosody is known to be an important ingredient in spoken language, not only expressing emotions but also potentially modifying, or even completely altering, the communicative meaning of an utterance (e.g., irony). Hellbernd and Sammler (2016) found that prosodic patterns of different speech acts, such as criticism, suggestion, or wish, were dissociable in terms of prosodic cues to a high degree and also consistent across speakers and utterances. We chose to focus on the prosodic modulation of utterances in the process of establishing meaning (see *meaning establishment* in Clark, 1994).

For this purpose, we developed the *prosodic comprehension of request for response* (PC-RR) test. In our test, participants judged whether an auditory utterance (e.g., *I don’t think your computer has the right port for this charger*) is meant as a simple statement (actors used few pauses, steadily falling intonation) or as soliciting feedback from the listener (actors paused between *has the right* and *port*, variable intonation shifting between falling and rising). Sentence materials were constructed so that the same literal material could be sensibly used in both conditions. We recorded 12 sentences where speakers (two male, two female) had been asked to produce prosody indicating a request for response from the listener, a request for response trial, or a statement trial. The sentences were presented to the participants whose task was to determine by button-press whether the sentence was a request for response or a statement trial. Each participant listened to all 12 sentences, half in the request for response condition, and half as statement sentences (order and mapping between condition and sentence were randomized). The test was implemented using PsychoPy (Peirce et al., 2019).

#### Production: Advanced audience design

While there are many psycho/neurolinguistic studies of production processes, they have always been outnumbered by comprehension studies, largely for methodological reasons (e.g., the issue of combining elicitation in production with experimental manipulation and control, as well as the issue of movement artifacts in neuroimaging). Perhaps the most common task that can be described as a pragmatic production task is the *director’s task*, which builds on an important insight from conversation analysis (Sacks & Schegloff, 1979)—interlocutors have to tailor each contribution to the specific needs of the listener by means of so-called *audience design* (Bell, 1984; Clark et al., 1983), alternatively called *recipient design* (Sacks & Schegloff, 1979).

In the director’s task, the participant produces labels for visually presented objects based on whether the objects are uniquely in their own field of view or seen mutually with the listener (Krauss & Glucksberg, 1977; Ryskin et al., 2015; Wardlow, 2013). The standard director’s task has, however, been criticized for possibly testing selective attention rather than pragmatic inferencing, as the participant may make use of the strategy to pay attention only to objects that are not occluded. This attentional strategy might for instance be implemented using shallow visual attentional mechanisms, as occluded/non-occluded objects have particular background colors (Rubio-Fernández, 2017). Another study showed similar performance when participants take the perspective of a camera instead of another person, which questions task validity when studying pragmatics (Santiesteban et al., 2015). To address these concerns, we designed a version of the director’s task where the speaker instead needs to consider the age, gender, and cultural background of the listener. In other words, the speaker needs to tailor their utterance to the specific addressee, based on their inferences about what the addressee knows. Validity is improved since it is the addressee’s *knowledge* that must be taken into account, just as in naturalistic communicative situations. The more participants take their knowledge of the listener into account, the higher their performance on the task. We thus consider this task a state-of-the-art task for measuring valid individual differences in AD, which was our goal in the context of the fMRI study. More generally, this new task probes how interlocutor characteristics (e.g., age) influence conversational processes.

Participants were instructed to describe an object that could not be assumed to be known in the *unknown condition* (test condition), or that could be assumed to be known in the *known condition* (control condition) to a fictitious addressee. An example is given in Table 1. A successful way of describing an object to the addressee in the unknown condition is to describe it in other, simpler words. Successfully paraphrasing the object in the test trial resulted in 1 point, while not paraphrasing (using a label that could not have been known to the addressee) gave 0 points.

**Table 1.** Examples of four trials in the audience design task for two different addressees

**Addressee**	**Test trial: Unknown condition**			**Control trial: Known condition**
	**Target**	**Example of paraphrasing**	**Competitor**	**Target**
6-year-old child	Corset	The beige thing that looks like a dress	Bikini	Banana
91-year-old person	Drone	The machine with four propellers	Helicopter	Recorder

*Note*. For the test trials, the competitor object and an example of a paraphrase indicating that the participant has succeeded in taking the addressee’s perspective are given. The two objects are shown in Figure S2. In this example, the object to be named was a corset (the competitor was a bikini), and when the addressee was a small child, an appropriate label would be *the beige thing that looks like a dress*, whereas a pragmatically inappropriate label would be *undergarment* since a child probably does not know what a corset’s function is, and bikini also fits this label.

Each trial consisted of an image of a bookshelf where five objects were placed along with a picture of the addressee specific to that trial. The *target* object was the object the participant had to describe. The *competitor* object (see Table 1) belonged to the same general category as the target object to make the participant avoid a more general description, which could be taken as a paraphrase. Three filler objects were also on the bookshelf. Each participant did 10 test trials and 10 control trials. The test was implemented using PsychoPy (Peirce et al., 2019).

### Behavioral Tests of ToM and Cognitive Control

#### Nonverbal behavioral ToM

An overall goal of the study was to delineate pragmatic processes that are potentially partly independent of more basic nonverbal ToM. We measured ToM from a standard nonverbal test of mentalizing ability: RMET (Baron-Cohen et al., 2001). Based solely on pictures of the eye region of human faces, participants had to attribute complex emotions to the pair of eyes by choosing from four alternatives. The test is independent of language abilities but related to the severity of ASD symptoms (Senju et al., 2002). We wanted to minimize the risk of a more trivial association between ToM and pragmatic ability, which might have resulted from some RMET items requiring the participants to resort to verbal strategies. We thus eliminated items where any communicative intention (i.e., sarcasm or joking) was cued. Out of the remaining trials we chose 12 in a way so as to maximize the variation of emotions and the difficulty of the test (using the correctness rates reported in Baron-Cohen et al., 2001). The test was implemented using PsychoPy (Peirce et al., 2019). Even though the RMET was originally intended as a test of ToM and has been extensively used as such, we note that there is now research rather linking it to the ability to recognize complex emotions (Oakley et al., 2016), somewhat independent of intention recognition. While emotions can be considered to be mental states and thus be part of ToM, Oakley et al. (2016) use Alexithymia and ASD patients to show that they are potentially segregated from domain-general ToM. Therefore, we refer to this task as a domain-general ToM/complex emotion recognition task in the discussion. It could thus potentially be viewed as reflecting “affective” rather than “cognitive” ToM (Shamay-Tsoory & Aharon-Peretz, 2007). Note, however, that there are known issues in measuring individual differences in ToM (Conway et al., 2019). This suggests that an alternative formulation of this part of the design is that we are testing the uniqueness of pragmatic processes relative to complex emotion recognition (or again, affective ToM). However, similar restrictive generalizability applies to other ToM tests.

#### Cognitive control – Operation span

The operation span (OSpan) test (Foster et al., 2015) has been designed to probe CCF, as well as working memory, as a part of CCF. The 199 participants in the behavioral experiment memorized a sequence of letters while resisting frequent distractors, as a test of their CCF. CCF, especially inhibitory control and working memory, are associated with performance on some pragmatic tasks, at least during development (Matthews et al., 2018).

The mixed adult literature includes Roßnagel (2000, 2004), who found that AD was negatively affected by higher cognitive load as induced by a dual task situation. Initial correlations between pragmatic processes and tasks measuring individual differences in general executive function and nonverbal IQ, such as the Stroop task, Raven’s matrices, and simple working memory span tasks, have been observed, while replication attempts have failed (Brown-Schmidt, 2009; Brown-Schmidt & Fraundorf, 2015; Ryskin et al., 2014). Ryskin et al. (2014) found a significant relation between a perspective-taking task and the OSpan task, providing at least one correlational result yet to be replicated or not.

Thus, again, as a measure of cognitive control, participants completed the OSpan test (Foster et al., 2015) as implemented in E-prime (Engle lab; https://englelab.gatech.edu/taskdownloads.html). In this test, participants are presented with a sequence of letters, one by one, and then asked to recall the sequence. Between each letter presentation, the participants must complete a distractor task in the form of a simple calculus problem (e.g., 3 * 4 + 1). For more details, see the supplementary material.

### Behavioral Tests of Language Ability

As indicated in the introduction, previous research has segregated pragmatic comprehension processes from core language ability, in the form of vocabulary and grammar. The participants in the behavioral experiment were assessed on core language tasks, and the fMRI participants on two additional tasks. These structural language measures were the AR test, which tests print exposure and predicts reading skill (Moore & Gordon, 2015) and cultural literacy (Stanovich & West, 1989; West et al., 1993); a vocabulary test taken from the Vocabulary, Swedish Scholastic Aptitude Test (vSweSAT of 2016; Cliffordson, 2004) an LDT (Holmer et al., 2016) measuring low level reading and lexical access processes. The vSweSAT and LDT were administered for the fMRI participants only. The vSweSAT consists of 10 words where the correct synonym is to be selected from five alternatives. There was no time limit to complete the test. What we refer to here as *core language skills* or *structural language skills* are actually a set of often interrelated subskills, commonly assessed and evaluated together in the clinical setting (see further section S1.4.1 in the Supporting Information).

#### Lexical decision task

In the LDT (Holmer et al., 2016), the participants were presented with 46 three-letter combinations (two consonants and one vowel) within a 5 s period, and were instructed to determine as fast as possible whether the combination was a real word (half of the trials) or not; order was randomized. Half of the words that were not real words were pseudowords, that is, they were orthographically legal but non-lexicalized items. The other half were non-words, that is, orthographically illegal items. For each participant, the reaction times for the correctly answered trials were averaged and multiplied by −1 to yield the final score where performance increases with score value.

#### Author recognition task

In the AR task, the participants were presented with a list of names in alphabetical order, half of which were real authors and half of which were not. The task was to identify which names were authors. A correctly selected author yielded 1 point, an incorrectly selected author yielded −1, and all other cases yielded zero. There was no time limit to complete the test. We adapted the AR task as given in Moore and Gordon (2015) to a Swedish reading audience and slightly revised the task by increasing the ratio of better known to lesser known authors in order to increase sensitivity to individual variation among participants with poor performance. This was advised by the authors in Moore and Gordon (2015) based on an item response analysis of their data. For more details, see the Supporting Information.

### Low/High Pragmatic Ability Groups

**Figure 1.**
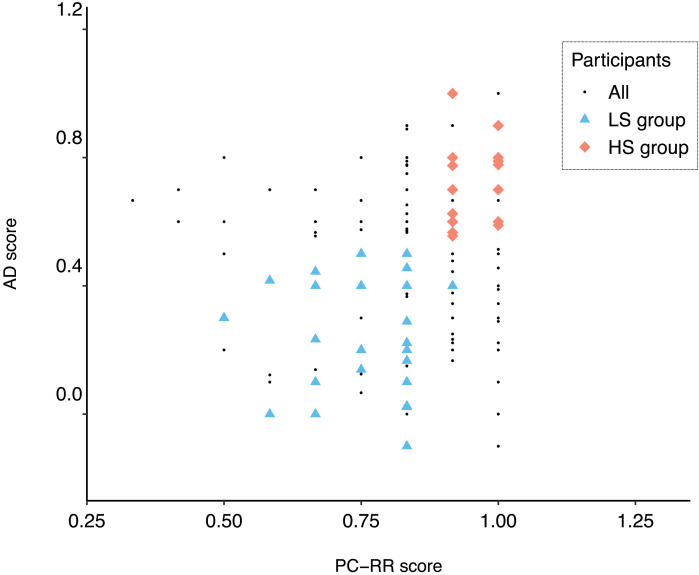
PC-RR and AD test scores for all participants. The participants in the high (low) score group are shown as red squares (blue triangles) and participated in the fMRI study. Participants who scored in the lower 50% on both the AD and PC-RR tests were placed in the LS group and those who scored in the higher 50% on both tests were placed in the HS group. Both of these groups include a wide range of scores within these limits, not only very low/high ones.

Our aim was to study individual variation in pragmatic ability in general, rather than performance on specific tests. We thus combined the different pragmatic tests of relevance to the fMRI paradigm, PC-RR and AD, and constructed two pools of participants, one with low and one with high pragmatic ability. The low (high) ability pool was formed out of those with scores lower (higher) than the mean on both the PC-RR and the AD tasks; see Figure 1. Participants in the fMRI experiment were then invited from these two pools, forming one low and one high ability participant group, the LS and HS groups. There were 41 participants in each pool, and participants were invited randomly until 30 in each group had been measured. In order to match the groups on cognitive control or complex working memory ability, as indicated by performance on the OSpan test, some participants from the pools were omitted.

Due to some further exclusion after measurements, the final LS (*N* = 29, 13 males) and HS (*N* = 28, 15 males) groups were somewhat smaller. One participant was excluded from the experiment after measurements due to excessive head movements (>4 mm) at several volumes. Another participant was excluded due to <80% correctness rate on the compliance questions (see below). Finally, one participant was excluded due to technical failure. The average scores and standard deviations of the PC-RR and AD tests across groups along with two-sample *t* tests of AD and PC-RR across groups are given in Table 2. While there were no significant differences between the two participant groups in terms of age, RMET, OSpan, or LDT, the HS group had significantly higher AR and vSweSAT scores (see Table 2).

**Table 2.** Average behavioral test scores, age, and gender for the two communicative skill groups

	**High score group**	**Low score group**	**Statistical tests**
PC-RR*	0.97 ± 0.04	0.74 ± 0.12	*t*(34) = 10.11, *p* < 0.001
AD*	0.70 ± 0.13	0.25 ± 0.18	*t*(50) = 10.62, *p* < 0.001
**RMET**	0.83 ± 0.12	0.82 ± 0.15	*t*(52) = 0.35, *p* = 0.73
AR*	0.14 ± 0.07	0.09 ± 0.05	*t*(53) = 2.58, *p* = 0.01
**LDT**	1,019 ± 190	1,026 ± 287	*t*(45) = −0.10, *p* = 0.92
vSweSAT*	0.86 ± 0.13	0.76 ± 0.20	*t*(45) = 2.22, *p* = 0.03
**OSpan**	0.72 ± 0.17	0.70 ± 0.16	*t*(55) = 0.55, *p* = 0.59
**Age**	29.97 ± 4.04	29.10 ± 4.97	*t*(52) = 0.71, *p* = 0.48
**Gender**	15 males	13 males	X(1) = 0.42, *p* = 0.52

*Note*. Average test scores and age (± standard deviation) for the two groups in the prosodic comprehension of requests for response (PC-RR) task, audience design (AD) task, reading the mind in the eyes test (RMET), author recognition (AR) task, lexical decision task (LDT), vocabulary scholastic aptitude test (vSweSAT), and operation span (OSpan) test along with statistical tests (Welch’s two-sample *t* test) of difference of these quantities between the groups. In the final row we report a statistical test (Pearson’s chi-square test with Yates’s continuity correction) of the difference in gender between the groups. An asterisk marks a significant difference in test scores between the groups.

### fMRI Task: The Indirect Speech Acts Experiment

#### Stimulus material

The stimulus material in the ISA task consisted of 78 experimental trials. These were short recorded dialogues: a question and a reply, preceded by a short context depicting the setting of the dialogue. There were two experimental conditions, *indirect* and *direct*; see Table 3. In the direct condition, the reply sentence was simply an informative answer to the question. In other words, the speaker meaning (see Introduction) coincided with the literal meaning. In the indirect condition, the literal reading of the reply is often or partly irrelevant (Grice, 1975) to the question. In these trials, the speaker meaning in the reply was different from, or included more meaning than, the literal meaning. While the literal content of the reply sentence was the same in both conditions, the context and question sentences varied, making the reply sentence direct or indirect (see Table 3). This was to ensure that any differences in neural activation between indirect and direct conditions observed during the reply would be a consequence of inferring the speaker meaning, rather than decoding the literal reply. To interpret the indirect replies, the participants thus had to make a pragmatic inference. In addition, these replies were affective in the sense that they “saved the face” of the addressee. According to face theory (Goffman, 1967), the *face* of a speaker can be defined as the “positive social value” that a speaker holds in relation to others in a communicative situation. In the indirect condition, the speakers saved the face by paraphrasing a message that would be face threatening if given in its explicit form (Brown & Levinson, 1987).

**Table 3.** Example of an ISA test trial in the direct and indirect version and a following compliance question

**Condition**	**Context**	**Question**	**Answer**
**Direct**	Magnus and Emilia are old friends. They are discussing how hard it is to find restaurants which both you and your partner fancy. Emilia asks Magnus:	Why doesn’t your girlfriend like Japanese food?	She is not so used to Asian seasoning.
**Indirect**	Magnus and Emilia are old friends. They are talking about the last time Magnus visited Emilia in her student dormitory. Emilia asks Magnus:	Did your girlfriend like my vegan noodles?	
**Compliance question**	Was there someone who was not so used to Asian seasoning?		
**Filler trial**	Benny and Ellinor are doing their laundry. Benny went out walking the dog but is now back. He asks Ellinor, who was the last to visit the laundry room.	Is the washing machine done?	Yes, just put everything in the dryer.

We focused on face saving speech acts as opposed to purely informative indirect replies based on previous research on ISAs in different social contexts (Holtgraves, 1998). Holtgraves found that for replies that seem to diverge from relevance (Grice, 1975), i.e., replies that do not explicitly (literally) answer the question, a face saving context made them more likely to be interpreted as ISAs. Half of the trials (39) delivered a critical opinion from the *speaker*, referred to as *Criticism* trials. The other half delivered a bad message or critical opinion from *another person* not present in the dialogue setting, referred to as *Bad message* trials. These two kinds of sentences were introduced to provide varied, yet controlled trial types. We did not have any specific hypothesis on how trial type would affect processing, and analysis across types was beyond the scope of the study.

We created 14 filler trials formatted like the other trials: a context, a question, and a reply. The replies were always direct, but in contrast to the replies in the indirect and direct trials, the filler replies were initiated by a *yes* or *no*. The filler trials were presented to make the pattern of indirect and direct answers less apparent. In order to make sure participants paid attention to the stimuli, they answered 12 compliance *yes*/*no* questions about the content of the previous trial; see Table 3. The questions regarded practical information that could be given in any part of the trial (context, question, or answer).

All participants answered with an accuracy of >83% on the compliance questions and had at most one error per run. There was no significant difference (*t*(59) = −0.76, *p* = .45) in the accuracy between the groups. No participants passed the far outlier threshold in terms of reaction times defined as the Q3 + 3 × IQR, where Q3 is the 75% percentile and IQR is the interquartile range.

Fifty-eight of the dialogues were revised and translated from Asaridou et al. (2019). We created 20 additional dialogues, as well as short contexts for all 78 dialogues. The number of words in the contexts and the questions was balanced across the two conditions (two-sample *t* tests, *p* > 0.73). Care was taken to make the contexts as similar as possible between the two conditions of each trial, as well as to use what we perceived as frequent words throughout. The average durations of the trial components are presented in Table 4. The intertrial intervals (ITIs) were sampled from a flat distribution between 2 and 4 s with bin size 0.1 s.

**Table 4.** Average durations of trial components [s]

**Condition**	**Context**	**Question**	**Answer**
**Indirect**	11.6 ± 2.5	2.9 ± 1.0	4.2 ± 1.0
**Direct**	11.6 ± 2.5	2.6 ± 1.0	3.8 ± 1.2

*Note*. Average durations ± standard deviations of context, question, and answer for the two conditions separately. No significant differences between the indirect and the direct conditions were found for the context, question, or answer.

#### fMRI procedure

Participants received scripted oral instructions about the ISA experiment. They were instructed to pay attention to what the protagonists “really intended to say.” The experiment was performed double-blind, such that neither the experimenters nor the participants knew which group (low- or high-scoring pragmatic ability) the participant belonged to.

The ISA task, with a total duration of approximately 38 min, was divided into 3 runs. Each run consisted of 26 unique experimental trials, 1–3 filler trials, and 1–5 compliance questions. Every participant was presented with 78 experimental trials (half indirect, half direct), 14 filler, and 12 compliance questions in total. Every experimental trial was presented in its direct vs. its indirect version to an equal number of participants (but a specific participant only heard any specific trial in either its direct or indirect version), to control for the exact literal meaning of each reply, across participants. For more details, see the Supporting Information.

#### fMRI and T1 data acquisition

The participants were scanned in an interleaved fashion with a Siemens 3T Magnetom Prisma MRI-scanner, using a 20-channel surface coil. Functional scans were acquired with a repetition time (TR) of 2.1 s and echo time (TE) of 30 ms; each volume consisted of 70 slices, 2.0 mm thick, with a 1 mm slice gap. The voxel size was 2.2 × 2.2 × 2.0 mm^3^ and the field of view was 210 mm. The flip angle was 70°. A whole-brain high-resolution structural T1-weighted magnetization-prepared rapid acquisition with gradient echo (MPRAGE) sequence (using generalized autocalibrating partially parallel acquisitions (GRAPPA) to accelerate acquisition) was performed to characterize a participant’s anatomy (TR = 2,300 ms, TE = 2.85 ms, 192 slices with 1.3 mm isotropic resolution, and field of view = 256 mm).

### Analysis of the Behavioral Data

The statistical analyses were performed using R (version 3.6.1; https://cran.r-project.org/bin/windows/base/old/). We tested for correlations between the AD and the PC-RR tests using a one-tailed Pearson correlation test. A one-tailed test was used due to the strong expectation that the two pragmatic tests would be positively rather than negatively correlated. As the scores of the behavioral tests were found not to be normally distributed as tested by the Shapiro-Wilk normality test, a rank based inverse normal (RIN) transformation (Bishara & Hittner, 2012) was performed on the data before testing the correlations with a Pearson product-moment correlation test (see further supplemental section S1.5).

### fMRI Data Analysis

The data were preprocessed and statistically analyzed with the statistical parametric mapping software SPM12 (https://www.fil.ion.ucl.ac.uk/spm/; Friston, 2007).

#### Preprocessing

We first performed motion correction (realignment), then coregistration of the functional images to the anatomical image, normalization to a standard Montreal Neurological Institute (MNI) space, and spatial smoothing. After realignment, the head movements in *x*, *y*, and *z* were checked independently. All participants had a head movement <3 mm, considering all directions. The normalization was carried out using affine regularization, and a resampling of the voxels to 2 × 2 × 2 mm was carried out using a 4th degree B-spline interpolation. During the normalization, white and grey matter segmentation and bias correction were also performed. The spatial smoothing was a 3D isotropic Gaussian smoothing kernel of full-width at half-maximum (FWHM) = 8 mm, applied to the functional data. We included a temporal high-pass filter (cycle cut-off at 128 s), to account for various low-frequency effects.

#### First level statistical analysis

We modeled the hemodynamic response function (HRF) for nine conditions separately and six motion parameters from the realignment using a general linear model (GLM). The first six (condition) regressors were context, question, and answer, for direct and indirect trials separately. The pause between a question and an answer was counted as part of the answer. We modeled compliance questions and the whole filler trials (i.e., speech of no interest) as one regressor. The response to the compliance question and the ITI (fixation cross) were both modeled separately. The regressors were convolved with a canonical HRF using a 2 mm within brain mask from the FSL software standard library provided in SPM12, including both white and grey matter but excluding ventricles. The (t) contrasts were (1) indirect vs. direct answer: indirect vs. direct, (2) indirect vs. ITI: indirect, (3) direct vs. ITI: direct, and (4) context vs. ITI.

#### Second level statistical analysis

Second level analyses were performed using contrasts defined at the first level. First, we evaluated the interaction between groups for indirect vs. direct contrast. (In the following, we will sometimes use only “interaction,” omitting the “between groups,” when referring to the second level analysis of interaction between groups.) We did this using a partitioned error one-way analysis of variance (ANOVA) between subjects with two levels: communication group and condition (indirect or direct), where the full model with the interaction is compared to the reduced model of only the main effect of condition. In Statistical Parametric Mapping software (SPM; https://www.fil.ion.ucl.ac.uk/spm/), this was implemented using the indirect vs. direct contrast from the first level, and these images were then entered in a two-sample *t* test with unequal errors at the second level. Following that, a [1 −1] F contrast was used to compute the interaction.

We applied a cluster-forming threshold of *p*_uncorrected_ = 0.001 (no extent-level threshold, *k* = 0). As a multiple comparison correction method, we used family wise error (FWE) correction at the cluster and peak level, as implemented into SPM. For this *F* test, we report all clusters with a *p*_FWE_ < 0.05. For each cluster we also report the test-statistic of the voxel (peak), if there is a peak with *p*_FWE_ < 0.05. We do not report any additional voxels, even if significant at *p*_FWE_ < 0.05.

We also compared the indirect and direct contrasts between the HS and LS groups using a two-sample *t* test with unequal errors and a [1 − 1] F contrast. In addition, we investigated indirect vs. direct for both groups together and for each group separately, using a one-sample *t* test (as implemented in SPM by a [1] T contrast).

As the literature reports lateralization in the lateral parietal lobe in the context of pragmatics (Ciaramidaro et al., 2007), we tested whether our left lateralized results were indeed significantly different from the corresponding region in the right hemisphere. To further investigate an activation in the parietal lobe (see Results), the average (across voxels in the cluster) indirect vs. direct first-level contrast values (i.e., signal values, not beta-values) from the cluster (and its mirror in the *y*–*z* plane) were thus extracted using Marsbar software (Brett et al., 2002). A paired *t* test between the left parietal cluster contrast values and the corresponding values in right regions was then carried out to test for significant lateralization.

To estimate the overlap between the clusters from the indirect vs. direct interaction between groups analysis and the language, ToM, MD/cognitive control networks, we tested for the number of overlapping voxels with neurosynth association masks for the terms *language comprehension*, *tom*, and *cognitive control*. For *cognitive control*, we also tested for overlap with the uniformity mask since the association mask was incomprehensive.

In a final analysis, we extracted the average indirect vs. direct first-level contrast values using the regions of interest (ROIs) defined by the significant clusters from the interaction analysis using Marsbar. The ROIs were the clusters formed with the threshold *p*_uncorrected_ < 0.001 (see sizes in Table 6(a)). These values were correlated with the scores on RMET, vSweSAT, and LDT for each participant (AR was not analyzed further, see the section: Behavioral results). We expected an absence of correlation for RMET and LDT as no significant difference between the groups in terms of RMET or LDT score existed. In contrast, there was a significant difference between the groups in vSweSAT performance and thus, we expected a correlation between activation data and the vSweSAT. As the behavioral data was not normally distributed, a RIN transformation (see the section: Analysis of the behavioral data) was performed before testing for Pearson correlation.

To determine the anatomic locations of the activations, the Harvard-Oxford Cortical Structural Atlas (https://identifiers.org/neurovault.collection:262) was used, occasionally complemented by Juelich Histological Atlas (https://fsl.fmrib.ox.ac.uk/fsl/fslwiki/Atlases/Juelich). Surface projection figures (trilinear interpolation) were created using the Connectome Workbench software (v1.5.0; https://www.humanconnectome.org/software/get-connectome-workbench).

## RESULTS

### Behavioral Results

Table 5 shows the results of the two-tailed Pearson correlation test on RIN-transformed data (see the section: Analysis of the behavioral data). The scores of the two pragmatic tests AD and PC-RR correlated significantly in the behavioral sample as tested by a one-tailed correlation test (again, a one-tailed test was used here because of the strong prediction that the two pragmatic tests would be positively rather than negatively correlated). Further, the PC-RR and AD scores both correlated significantly with the AR test and the vSweSAT test using a two-tailed test, but not with the LDT. The PC-RR correlated significantly with the OSpan test, while the AD test did not. None of the pragmatic tests correlated with the RMET. The AR and vSweSAT tests both correlated significantly with the OSpan test scores, and RMET correlated significantly with the LDT; see Table 5. Finally, the two language tests AR and vSweSAT correlated significantly. AR was dropped from further analysis for this reason and because we were more interested in a test of vocabulary, as it is a central, well-researched core language ability (it is less clear what exactly AR tests).

**Table 5.** Pearson correlation tests on RIN-transformed data for the behavioral tests

	**Behavioral test score correlations**						
	**Pragmatic tests**		**CCF**	**ToM**	**Language tests**		
	**AD *N* = 194**	**PC-RR *N* = 198**	**OSpan *N* = 198**	**RMET *N* = 193**	**AR *N* = 198**	**vSweSAT *N* = 60**	**LDT *N* = 58**
AD	–	*p* = 0.039 (one-tailed)	n.s.	n.s.	*p* = 0.010	*p* = 0.012	n.s.
PC-RR		–	*p* = 2.9 · 10^−3^	n.s.	*p* = 8.2 · 10^−3^	*p* = 4.6 · 10^−4^	n.s.
OSpan			–	n.s.	*p* = 0.042	*p* = 5.6 · 10^−4^	n.s.
RMET				–	n.s.	n.s.	*p* = 0.024
AR					–	*p* = 9.2 · 10^−6^	n.s.
vSweSAT						–	n.s.

*Note*. All *p* values and correlation coefficients can be found in Table S2. n.s. stands for nonsignificant (*p* > 0.05). All tests are two-tailed except for the AD and PC-RR correlation. No correction for multiple comparisons was applied. We refrain from drawing any conclusions from the Pearson correlation coefficients themselves, which is why we are not showing them here (see further discussion in the section: Analysis of the behavioral data).

### fMRI Results

#### Indirect vs. direct contrast

**Figure 2.**
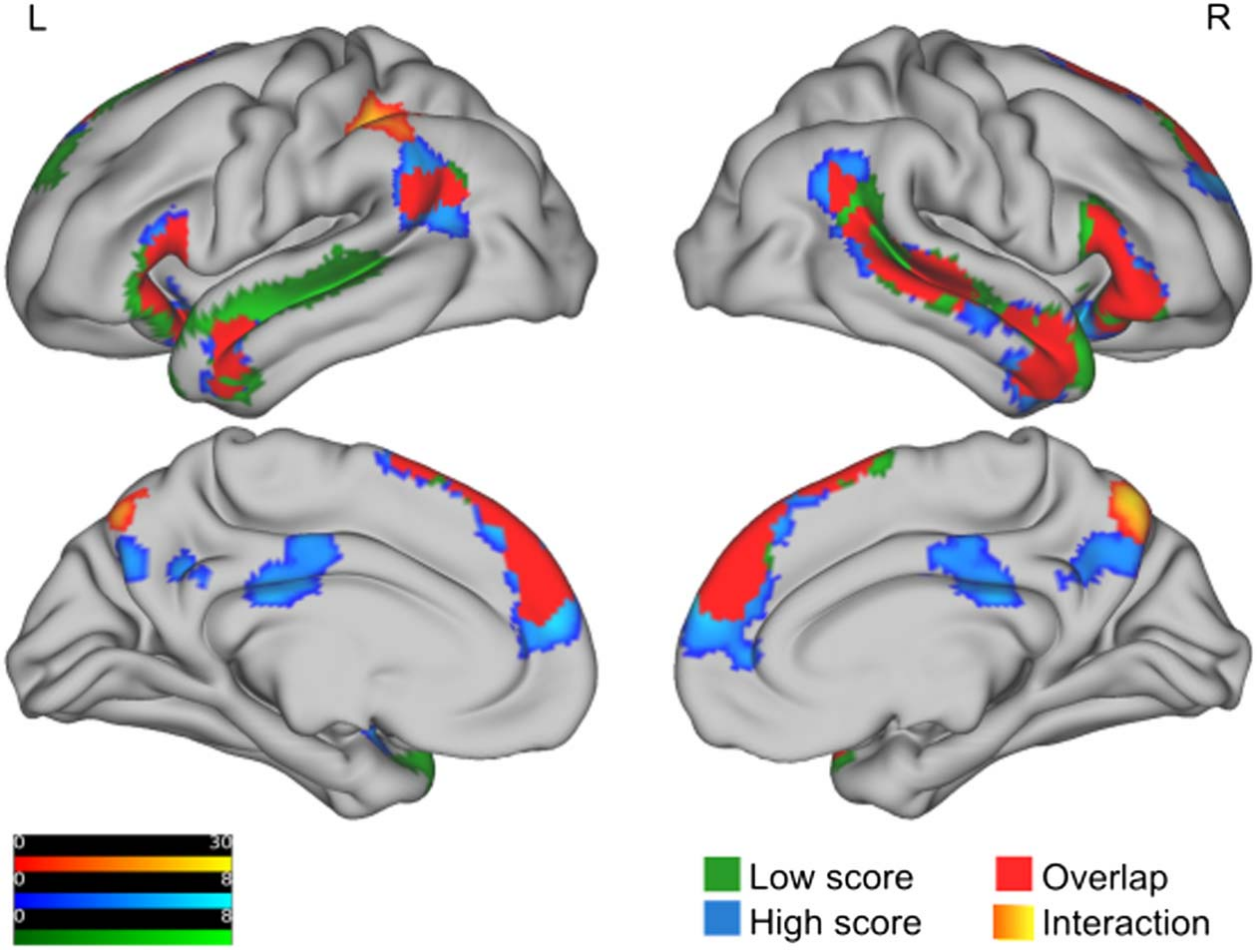
Brain activation for the indirect vs. direct contrast, for each group separately as well as for the interaction between the groups. The figure shows *F* values for clusters with a cluster-forming threshold of *p*_uncorrected_ = 0.001 (no extent-level threshold, *k* = 0). As a multiple comparison correction method, we used family wise error (FWE) correction at the cluster and peak level. We only report clusters and voxels with a *p*_FWE_ < 0.05. All clusters are projected onto a pial surface. The activation for the interaction is shown in orange to yellow. The activation for the high (low) score group is shown in blue (green). The regions where the activations in both groups overlap are shown in solid red and no activation information is given. Note that the range of the scales do not reflect the exact max (min) value of the unit of activation (which is customary) but are adjusted slightly so as to include values from all hemispheres.

We report the indirect vs. direct contrast for both HS and LS groups in Table 6 and for each group separately in Table 7. We observed overlapping activity for the HS and LS groups in bilateral IFG, TPJ, anterior temporal lobe, medial superior frontal gyrus (SFG)/dorsomedial prefrontal cortex (dmPFC), and right mid and posterior MTG/superior temporal sulcus (STS; see Figure 2). In addition, the LS group showed left posterior MTG/STS activity. The HS group activated a larger portion of the same cortices, ventrally and dorsally in the left TPJ, and ventrally/rostrally in the medial SFG/dmPFC. This group also showed significant activity (absent in the LS group) in the left cerebellum, bilateral posterior cingulate, and precuneus, a pattern which resulted in a significant group interaction for only the precuneus cluster. Note that differences across groups that are evident in this latter kind of analysis are not straightforward to interpret as they are not statistically tested interactions with groups (for this see instead the section: Indirect vs. Direct contrast – Interaction between the HS and LS groups). In Figure 2, we also report some of these analyses for illustrative purposes.

**Table 6.** Activations for contrast indirect vs. direct, interaction between groups, and for both groups

**Anatomical region**	**MNI local maxima**			**Cluster**		**Voxel**	
	***x***	***y***	***z***	**Size**	***p*_FWE_**	***t* / *F* value**	***p*_FWE_**
(a) Indirect vs. direct interaction between groups (*F* test)							
Left superior parietal lobe/left SMG	−40	−48	46	368	<0.001	n.s.	
Bilateral precuneus	8	−66	54	399	<0.001	n.s.	
(b) Indirect vs. direct between groups HS > LS (follow up *t* test for direction)							
Left superior parietal lobule/left SMG	−40	−48	46	732	<0.001	*t*(55) = 5.37	0.040
Bilateral precuneus	8	−66	54	525	<0.001	n.s.	
(c) Indirect vs. direct between groups LS > HS (follow up *t* test for direction)							
No significant clusters or voxels							
(d) Indirect vs. direct for both groups							
Right MTG, angular gyrus, temporal pole, and IFG/frontal orbital cortex	50	28	−4	5642	<0.001	*t*(56) = 9.38	<0.001
Bilateral superior frontal gyrus	8	50	40	4070	<0.001	*t*(56) = 8.93	<0.001
Left cerebellum	−26	−82	−36	418	0.001	*t*(56) = 6.90	<0.001
Left MTG, angular gyrus and IFG/frontal orbital cortex	−60	−50	26	4534	0.001	*t*(56) = 6.88	<0.001
Bilateral precuneus	6	−54	36	438	<0.001	n.s.	
(e) Indirect group comparison							
No significant clusters or voxels							
(f) Direct group comparison							
No significant clusters or voxels							

*Note*. See the section First level statistical analysis for detailed explanations of the contrasts. The cluster-forming threshold was *p* = 0.001. Coordinates are given in MNI space. n.s. stands for nonsignificant.

**Table 7.** Activations for contrast indirect vs. direct for both groups separately

**Anatomical region**	**MNI local maxima**			**Cluster**		**Voxel**	
	***x***	***y***	***z***	**Size**	***p*_FWE_**	***t* / *F* value**	***p*_FWE_**
(a) Indirect vs. direct for HS group							
Right M/STG	54	−24	−12	1,465	<0.001	*t*(28) = 7.94	0.001
Right SFG/anterior paracingulate gyrus	6	62	24	2,969	<0.001	*t*(28) = 7.65	0.002
Left angular gyrus/occipital cortex/pSMG	−62	−54	28	937	<0.001	*t*(28) = 6.85	0.009
Right frontal orbital cortex/insula	30	18	−14	205	0.026	*t*(28) = 6.81	0.010
Right IFG/frontal pole	48	32	−2	669	<0.001	*t*(28) = 6.62	0.015
Right temporal pole	48	10	−32	583	<0.001	*t*(28) = 6.13	0.044
Left frontal orbital cortex/insula	−28	14	−18	774	<0.001	*t*(28) = 6.07	0.041
Bilateral posterior cingulate	0	−26	24	375	0.001	n.s.	
Left cerebellum	−32	−86	−34	330	0.003	n.s.	
Bilateral precuneus	−4	−68	34	387	0.001	n.s.	
Left temporal pole	−54	6	−28	218	0.020	n.s.	
(b) Indirect vs. direct for LS group							
Right frontal orbital cortex/IFG/temporal pole/STG/MTG	48	26	−8	3,339	<0.001	*t*(27) = 8.69	<0.001
Bilateral SFG	−6	54	24	1,920	<0.001	*t*(27) = 7.37	0.004
Left frontal orbital cortex/IFG	−50	26	−6	1,736	<0.001	*t*(27) = 7.00	0.009
Left angular gyrus/SMG	−60	−56	16	272	0.003	n.s.	

*Note*. See the section First level statistical analysis for detailed explanations of the contrasts. The cluster-forming threshold was *p* = 0.001. n.s. stands for nonsignificant.

#### Indirect vs. direct contrast – Interaction between the HS and LS groups

Our main analysis testing for interaction between groups for the indirect vs. direct contrast with an *F* test revealed two significant clusters; see Figure 2 and Table 6. One significant cluster was found in the parietal lobe, with parts in the superior part of the posterior as well as the anterior division of the supramarginal gyrus (SMG). Slightly more medial parts extended into the superior parietal lobe, where anterior parts connected to the postcentral gyrus and posterior parts connected to the angular gyrus (and anterior and mid-parts of intraparietal sulcus (IPS); Juelich Histological Atlas (https://fsl.fmrib.ox.ac.uk/fsl/fslwiki/Atlases/Juelich)). This cluster was adjacent to significant activity in the lateral left inferior parietal cluster that was only present for the HS group for the indirect vs. direct contrast, but it was located at the superior bank of this cluster (see Figure 2). Another significant cluster was found in the lateral parts of the right precuneus, extending into the left precuneus. A follow-up *t* test revealed that these two activations were stronger for the HS group compared to the LS group (see Table 6). Note that, as we selected the *F* test for analysis, this analysis was performed to find out the direction of the effect in the cluster, referred to in the section: Indirect vs. direct contrast – Interaction between the HS and LS groups, that was found in the *F* test only (i.e., it is not a parallel but a subordinate test). A lateralization test of the parietal cluster showed the lateralization not to be significant: A paired *t* test of the blood oxygen level-dependent (BOLD) responses from the indirect vs. direct contrast, between the left parietal cluster and corresponding right regions, was nonsignificant across all participants (*p* = 0.34) and in the HS (*p* = 0.48) and LS (*p* = 0.35) groups separately.

#### Context vs. ITI contrast

Using an *F* test identical to the one reported above (in the section: Indirect vs. direct contrast – Interaction between the HS and LS groups), we found no significant group differences (*p* > 0.88) during listening to dialogue contexts. This *p* value refers to the smallest FWE-corrected *p* value from a pool of *p* values at both the cluster and peak levels. Thus, our groups did not differ in semantic or structural processing at the level of sentences or mini-discourses (maximal number of sentences in a context is 4).

#### Overlap between indirect vs. direct interaction between groups result, and the language, ToM, and MD/cognitive control networks

Table 8 presents the results of the analysis described in the section Second level statistical analysis, where neurosynth masks were used to estimate the overlap between the core language, ToM, and MD/cognitive control networks on one hand and the parietal and precuneus cluster found in the indirect vs. direct interaction analysis, on the other hand. The overlap was generally low or non-existing, except for the uniformity cognitive control mask, which showed an 82% overlap with the parietal cluster.

**Table 8.** Overlap between indirect vs. direct between-group interactions and networks

**Cluster (total number of voxels)**	**Number of overlapping voxels per neurosynth mask**			
	**Language comprehension (association)**	**ToM (association)**	**Cognitive control (association)**	**Cognitive control (uniformity)**
Parietal (368 voxels)	0/368 = 0%	0/368 = 0%	7/368 = 2%	301/368 = 82%
Precuneus (399 voxels)	0/399 = 0%	3/399 = 1%	0/399 = 0%	36/399 = 9%

*Note*. The number (and percentage) of voxels from each cluster in the indirect vs. direct interaction analyses (rows) that lie in each neurosynth mask (column). Note that *cognitive control* was used as a term to create a network corresponding to the MD network.

#### Correlation analyses for the indirect vs. direct contrast vs. behavior

**Figure 3.**
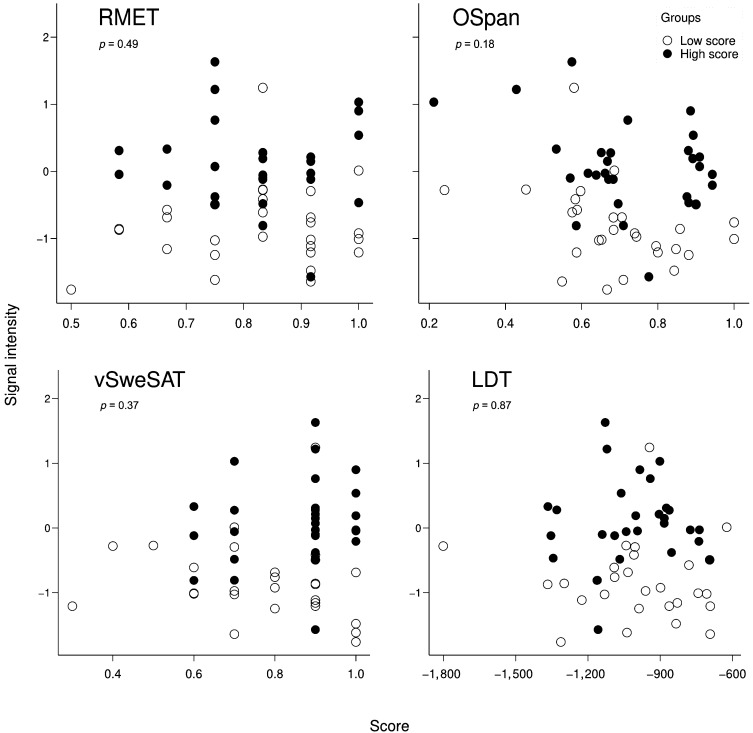
Parietal cluster. Single subject signal average (across voxels in the cluster) of indirect vs. direct first level contrast values for the parietal cluster vs. scores for four behavioral tests: RMET, OSpan, vSweSAT, and LDT. Data for participants from the high (low) group are shown as filled (not filled) dots. The *p* values indicate the outcome of two-tailed Pearson correlation tests with RIN-transformed data.

**Figure 4.**
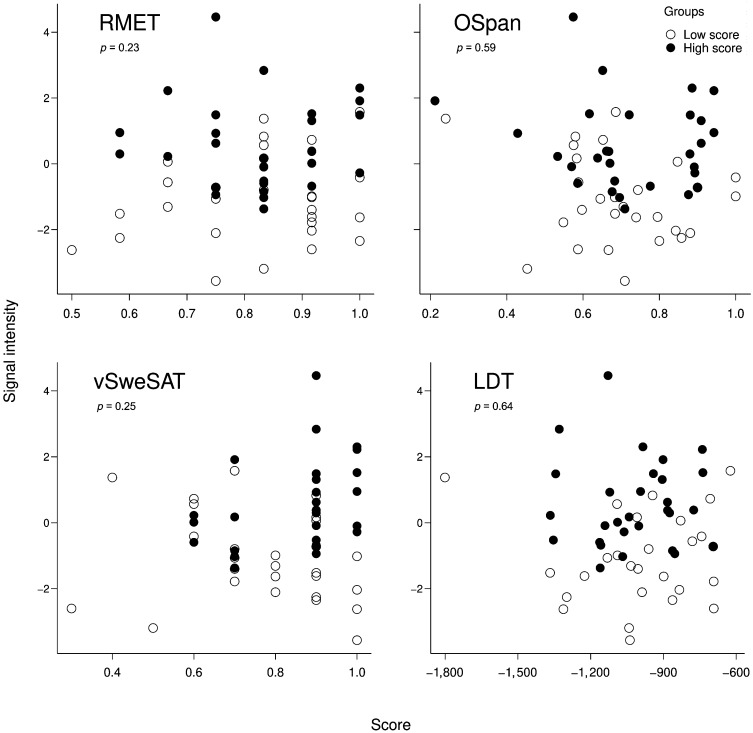
Precuneus cluster. Single subject signal average (across voxels in the cluster) of indirect vs. direct first level contrast values for the precuneus cluster vs. scores for four behavioral tests: RMET, OSpan, vSweSAT, and LDT. Data for participants from the high (low) group are shown as filled (not filled) dots. The *p* values indicate the outcome of two-tailed Pearson correlation tests with RIN-transformed data.

As previously described in the section Second level statistical analyses, indirect vs. direct contrast values, extracted from the interaction analysis clusters (the parietal cluster and the precuneus cluster), were plotted against and tested for correlation with, the behavioral scores of RMET, OSpan, vSweSAT, and LDT (see Figures 3 and 4, as well as Figure S4). This revealed no significant correlations for RMET, OSpan, vSweSAT, or LDT; see Figures 3 and 4.

The results for RMET, OSpan, and LDT were expected as no significant difference between the groups in terms of RMET, OSpan, or LDT score existed. In contrast, there was a significant difference between the groups in vSweSAT performance, and thus, the absence of correlation between activation data and the vSweSAT score is perhaps more unexpected and more informative.

## DISCUSSION

In an established paradigm comparing indirect vs direct speech acts *across all participants*, we report our first results as expected activations in core language regions and beyond. Interestingly, individuals belonging to a group with high communicative skills (the HS group) showed significantly more activity than those in a group with a low communicative skills (the LS group) in two cortical clusters: (1) left superior and inferior lateral parietal cortex, and (2) bilateral dorsal precuneus (see Figure 2 and Table 6). These regions are located outside core language and ToM regions (neurosynth language comprehension and ToM association maps; see Table 8). Their activity did not depend on either of the core language skills (speed of lexical access and vocabulary) or on ToM skills (see Figures 3 and 4). The absence of correlations with the vocabulary test are noteworthy since behaviorally, our two pragmatic tests did correlate with this test, which is not the case for the ToM test (Table 5). There was no effect of high vs. low communicative skill when participants initially listened to the informative context, which strengthens the specificity of the results to making communicative inferences. We suggest that complex aspects of intention processing are subserved by a network including these two regions (and potentially additional regions as discussed below). Based on the ToM related results (behavioral correlations, neuroimaging segregation, and fMRI vs. behavioral correlation), these complex aspects of intention processing may be specific to communicative situations. Importantly, our results show a neurocognitive separation between these communicative inferences and comprehension processes that are rather involved with, for example, lexical and sentence level semantic and syntactic processing of the literal material, which are subserved by different regions in the core language network.

### The Indirect vs. Direct Effect, in Both Groups, as Potential Low Level Pragmatics

We verified that the activation pattern for the indirect vs. direct contrast for both groups replicated the results in previous studies with similar paradigms (Asaridou et al., 2019; Bašnáková et al., 2014); see Figure 2, Table 6, and Table 7. The same basic pattern was also present for the HS group and LS group, although there were visible differences between HS and LS, e.g., in the lateral left inferior parietal region and the medial prefrontal cortex. The interpretation of our reported individual differences rests on this replicability of the basic contrast of passive observation of indirect vs. direct speech acts across different versions of the ISA experiment that has been observed in the literature, for example, across modalities (Asaridou et al., 2019; Bašnáková et al., 2014). In this basic contrast, we observed overlapping activity for the HS and LS groups in bilateral IFG, TPJ, anterior temporal lobe, medial SFG/dmPFC, and right mid and posterior MTG/STS (see Figure 2). The HS group activated a larger portion of cortices, ventrally and dorsally, of the left TPJ. This group also showed significant activity (absent in the LS group) in the precuneus, a pattern which partly resulted in a significant group interaction. Essentially, all areas reported in the HS and LS groups, separately or in the overlap between groups, were thus part of either the neurosynth language comprehension or ToM association maps, in sharp contrast to the areas in the interaction that showed no overlap with these maps, as reported in Table 8.

There are many possibilities of what these frontotemporo and frontoparietal regions revealed by the indirect > direct contrast, might reflect. One line of explanation is to simply see them as reflecting core language processes and ToM processes that are recruited more for the indirect vs. the direct condition. The frontotemporal regions overlapping with the neurosynth language comprehension map could then correspond to top-down (downstream) effects of pragmatic processing on core language processing. For instance, there is evidence that listeners recognize certain speech acts similar to ISAs early on in utterance (Gisladottir et al., 2015), which would allow for enhanced literal (or ultimately sentence level) processing, as a downstream consequence of an ISA. It should be noted that by design, these activations cannot, however, correspond to bottom-up literal processing (bottom-up here relative to pragmatic processes). The frontoparietal regions overlapping with the ToM association maps, on the other hand, could correspond to intention processing, perspective-taking, and other ToM processes. A second line of explanation is that both these frontotemporal and frontoparietal regions reflect processes such as the construction of a situation model, as the indirectness introduces a relative complication in the situation model (Heidlmayr et al., 2020). More generally, these areas could reflect low-level pragmatic processing, whereas the interaction would reflect more high-level pragmatic processing.

### Interpreting the Group Interaction: Pragmatics vis-à-vis ToM and Cognitive Control Functions

Recall that the HS group showed higher indirect vs. direct activity than the LS group in the left lateral parietal cortex and dorsal precuneus, outside core language and ToM regions (see Figure 2 and Tables 6 and 8). Note that this group difference is present despite an absence in other parameters (see Table 2): There were no group differences in age, gender, behavioral ToM skill (RMET), and CCF (OSpan). Furthermore, there was an absence of group differences in processing the informative context, and no differences in performance of comprehension questions.

As reported in Table 8, one of the neurosynth masks created by the *cognitive control* search term did have a large overlap with the superior/inferior lateral parietal cluster. Additionally, based on other reports in the literature on the location of the relevant networks, it would be possible to consider the superior/inferior lateral parietal cluster to be roughly located in the overlap of the ToM network and the MD network (Vincent et al., 2008). Likewise, the dorsal precuneus cluster could be considered a ToM area (see further discussion). However, it is notable that the complete ToM or MD networks were not activated. Thus, considering the locational information alone, activity in the two reported clusters could reflect MD/cognitive control processes. There was, however, no overlap with the neurosynth ToM masks and the two clusters. There is additional evidence of segregation from the behavioral results (see Table 5) as the performance on the ToM task (RMET) did not correlate with any of the pragmatic tasks (PC-RR or AD). For CCF, the behavioral data shows that even though there seems to be an interrelation between the OSpan test and the prosodic comprehension test (PC-RR), there was a nonsignificant correlation with the AD production task. When we used the individual variation in the RMET behavioral task as a proxy for variation in ToM as a neurocognitive system, we found that neither of the two regions reported were modulated by individual differences in ToM/complex emotion recognition (see Figures 3 and 4). The behavioral results follow the same pattern, and thus the absence of modulation was expected due to the absence of difference between the two groups (similar reasoning applies to the OSpan task). Taken together, these results suggest a potential neurocognitive segregation of domain-specific communicative inference from domain-general ToM/complex emotion recognition.

### Interpreting the Group Interaction as Potential Higher Order Pragmatics

As for sketching the nature of the potentially higher order pragmatic processing in the precuneus and parietal areas in the interaction between groups, we suggest that complex aspects of intention processing (see further the sections: Interpreting the group interaction: Pragmatics vis-à-vis ToM and cognitive control functions; Precuneus findings and large-scale functional networks; Inferior and superior parietal cortex) and related internal and external attentional processes (see further sections: Inferior and superior parietal cortex; Intraparietal sulcus, attention and intention) are subserved by these two regions (and potentially additional regions). Again, the ToM part of the results suggests some specificity of these intentional and attentional processes to the communicative situation. While communicative intention is fairly well explored in the literature (Brunetti et al., 2014; Enrici et al., 2019; Rasgado-Toledo et al., 2021; Tettamanti et al., 2017), there is less written about attentional processes specific to the communicative situation, but we suggest that they might entail contextually driven attention to verbal and prosodic cues that would reveal an indirectness in an utterance, even before these cues occur. Importantly, the absence of an interaction with groups when listening to the contextual information, as well as the absence of an interaction with group performance on the comprehension questions argues against group differences in terms of paying bare attention (as opposed to attention to, e.g., specific cues) to the stimuli or comprehending the literal content. Because of the overlap of the superior parietal cluster in the interaction between groups and the cognitive control uniformity neurosynth map, we cannot exclude the possibility that these areas represent more general cognitive control processes (attentional processes can again be mentioned as a possibility). (See further sections: Interpreting the group interaction: Pragmatics vis-à-vis ToM and cognitive control functions; Inferior and superior parietal cortex; Intraparietal sulcus, attention and intention.)

### Precuneus Finding and Large-Scale Functional Networks

Unlike the parietal cluster we observed, the precuneus is a highly connected hub area (Nijhuis et al., 2013). As such, it should not come as a surprise that it has been implicated in several large-scale functional networks. First of all, it is often considered part of a general ToM network (Paunov et al., 2019), but not always (Schurz et al., 2014), as is clear from its absence from the ToM neurosynth mask used in Table 8. It has also been proposed as the very center of a suggested intention processing network (Enrici et al., 2019). Our finding of increased precuneus activity for participants with high pragmatic skills indicates that this area is indeed an important cortical node subserving communicative intention processing and more specifically, as in our task, the discernment of different speech acts. However, second, while the precuneus is only sometimes included in the ToM network, it is a core node in the default mode network (DMN), and thus is a possible interface (and sometimes overlaps) between the ToM and DMN (Amft et al., 2015; Mars et al., 2012). Third, precuneus has been implicated in the (right lateralized) FPCN. We will come back to these observations when we discuss the parietal findings in the following two paragraphs, from two different perspectives.

### Inferior and Superior Parietal Cortex

The finding of increased activity for high skill participants indicates that this area is indeed an important cortical node subserving communicative intention processing.

This suggests that previous views on intention processing in lateral parietal and temporal areas might have to be modified. For instance, in an overview of the intention processing network, Enrici et al. (2019) suggest that the precuneus, right TPJ, and the mPFC—all three activated in our group averages (Figure 2)—encode “prospective social intentions,” while these regions plus the left TPJ encode communicative intentions. The left TPJ is thus the region they suggest to be specifically involved in communicative intentions; note, however, that the suggestion of specific intentional roles (e.g., prospective social intentions) is only empirically supported by one study with a small sample size of *N* = 12 (Ciaramidaro et al., 2007). Our results suggest that the literature should not ignore the intention processing role played by lateral parietal areas dorsal of the TPJ (in addition to the precuneus, TPJ, and mPFC), as activity in these areas may speculatively indicate routine recruitment linked to successful pragmatic performance. As a comment on lateralization, with respect to this region, it is important to first note that in a direct test of asymmetry, the right hemisphere area corresponding to the left lateralized cluster we report does not show a significantly different pattern (we discuss this further in the supplemental discussion).

Turning to the inferior parietal cortex, specifically the angular and SMG, these areas were activated by both groups (see Figure 2), while the group interaction was in the superior parts of these regions (extending into the IPS; see the next paragraph). The most likely interpretation is that activity in the angular gyrus reflects conceptual processing of (indirect) speech acts, given its presumed role as a main hub for conceptual/semantic processing (Binder et al., 2009).

### Intraparietal Sulcus, Attention, and Intention

Given our results, in particular the findings in the mid and anterior IPS, it is relevant to also discuss two additional large-scale brain networks that we did not choose to focus on a priori. These networks are (1) the dorsal attention network (DAN), including the bilateral IPS, superior parietal cortex, precuneus, and the frontal as well as supplementary eye fields; and (2) the mirror-neuron system (MNS), including the bilateral anterior IPS, premotor cortex, and sometimes inferior frontal cortex. The IPS is included in the top-down DAN. A specific suggestion has been made for the domain-general attentional function of IPS. According to Bisley and Goldberg (2010), the IPS consists of a set of priority maps selecting relevant features (hence a top–down attentional process) across different tasks. This kind of explanation has also been suggested in the context of a language task (Kristensen et al., 2013). From this point of view, the IPS activity of the high score communicators might be providing more relevant top–down attentional guidance, in the form of more precise and goal-effective priority maps, e.g., to turn attention to the potential verbal and prosodic cues that reveal the indirectness in the target utterance, before they occurred in the stimuli. According to the view of Vincent et al. (2008), our findings in the parietal cortex (including lateral inferior and superior parietal cortices and the IPS) lie at the intersection of the DAN and the FPCN (or MD) network, while our precuneus findings lie at the intersection of their FPCN and the DMN network. A possible interpretation is that these findings reflect the HS group’s better external and internal attentional control, respectively. We cannot exclude that the HS group found the task easier, which might have enabled a larger amount of mind-wandering, potentially activating the DMN. However, it is then unclear why only this part of the DMN (rather than the whole network) would be activated.

For the action system, Hamilton and Grafton (2006) suggest a hierarchical model of motor regions with the anterior IPS at the top. This area is thought to represent action goals and intentions. While this suggestion is based on a large literature of hand-actions (Hamilton & Grafton, 2006; Iacoboni et al., 2005), e.g., grasping actions and tool use, the question is whether this analysis has any relevance for speech actions that we make during conversation. A reasonable amount of skepticism is needed as the study of the MNS has suffered from severe hype (Hickok, 2009), making the literature hard to navigate. Some authors interested in language, communication, and other forms of joint action have indeed asked the question (Brennan et al., 2010) or more directly suggested that the MNS is also involved in these tasks (Bekkering et al., 2009). Does increased IPS activity for good communicators correspond to increased intentional processing? The explanation is simple enough as stated, but there are also several comments that need to be made alongside this suggestion. First of all, if there is any truth to this statement, we would like to decouple this interpretation from the MNS, as we do not observe any activity in other regions interpretable as mirror-neuron regions. If the anterior IPS is indeed at the top of the action hierarchy, encoding intentions and goals, then this model is worth also considering when studying conversational processes, but we do not see any reasons for further reference here to mirror-neurons. Second, a limitation for this interpretation is that the IPS is not routinely observed in communicative paradigms. For instance, it is not observed in many of the studies using versions of the ISA experiment (Asaridou et al., 2019; Bašnáková et al., 2014), although the left anterior IPS was observed in Egorova et al. (2016), who contrasted request vs. naming, only using direct speech acts (see also van Ackeren et al., 2016). It is possible that the individual differences approach improved sensitivity for the very top of the hierarchy, which might not be as activated in all participants and hence not detected under conditions of group averaging. In summary, we here provide possible attentional and intentional interpretations of the unexpected IPS activity.

### Theoretical Considerations

Our results can be discussed in the light of the theoretical suggestion of a submodule for communicative inferences (Sperber & Wilson, 2002). In Sperber and Wilson’s original suggestion from relevance theory, inferential processes in comprehension are subserved by dedicated domain-specific mechanisms, constituting a module (Fodor, 1983). Our results suggest that pragmatic processes are likely mainly instantiated with mechanisms distinct from core language mechanisms, as well as distinct from domain-general ToM mechanisms, even though we investigate a broader set of pragmatic processes than originally suggested by Sperber and Wilson (2002). Instead of referring to mentalizing as a unitary process, it is an interesting suggestion that mentalizing is a large and multidimensional space of mental state representations, a “mind space,” which would dynamically inform any situation where there is a need to consult somebody’s mental state (Conway et al., 2019). This is a promising future direction for ToM research.

Note that our findings do not allow us to take a stance on whether pragmatics constitutes a (weak) module or a submodule of ToM, as suggested by relevance theory. Our position is that pragmatics is neurocognitively separable from core language as well as (at least partly) from aspects of ToM, and that the concept of ToM will need differentiation. In other words, pragmatic inferencing does not equal mentalizing. We endorse the approach to study pragmatics in its own right, which should continue.

Pragmatic analyses of different types of utterances even suggest that not all interpretations of pragmatic phenomena require access to the mental state of the speaker in the first place, as is the case of some generalized conversational implicatures pointed out in the introduction. Some authors argue that even certain ISAs, such as indirect requests (e.g., *It’s cold in here*) can be interpreted without necessarily considering the mental state of the speaker (Kissine, 2016). Rather, the interpretive process can sometimes be based purely on the accessibility of certain salient information in the context; for example, the listener knows that closing an open window will relieve the speaker’s unpleasant feeling of being cold (see further the effort/effect trade-off used in these inferences, below).

Another perspective from relevance theory (Sperber & Wilson, 1996) is that optimal relevance is a trade-off between *cognitive effort* and effect on the listener. In cases of ambiguity “hearers tend to choose the most salient or accessible meaning, the one that costs the least processing effort to construct” (Sperber & Wilson, 2002, pp. 6–7; see also Gernsbacher, 1995). For them, this shows that communicative meaning is not coded, but inferred outside the explicit and conventionalized linguistic code system. Cognitive effort is thus suggested to be at the very heart of processes establishing meaning. This effort might of course partly, or even mainly, be related to pragmatic processes, but there is no particular reason why limited domain-general capacities, e.g., limited CCF, would not also produce similar effects on inferential processes. We provide some evidence in this direction, as our behavioral comprehension measure PC-RR correlated to a robust significant degree with OSpan in the behavioral study (*N* = 198), while the production AD task did not. This finding has clinical relevance (Cummings, 2015).

### Neurobiological and Clinical Perspectives

From a neurobiological perspective we provide evidence of two clusters constituting part of a network with the function of interpreting communicative meaning: left superior and inferior lateral parietal cortex (including IPS) and the bilateral dorsal precuneus. Individual differences in our ISA experiment are related to other pragmatic processes, which is also informative for future research in neuropragmatics. More specifically, a correlation between the two pragmatic measures of prosodic speech act comprehension and AD in production was found behaviorally (note, however, the one-tailed threshold and absence of multiple comparison corrections for this test; see Table 5). A group interaction analysis revealed significant activation during the ISA task. Since the groups were formed based on the pragmatic tests, this result suggests (albeit somewhat indirectly) a relation between three different pragmatic processes, one in production (AD) and two in comprehension (PC-RR and ISA), together suggesting that contextualized (and multimodal) communication requires interrelated skill sets. These results, including the neural results, are relevant for clinical research, for instance, in relation to the more recent and specific diagnoses pragmatic language impairment (4th ed.; *DSM-IV*; American Psychiatric Association, 2000) and social (pragmatic) communication disorder (5th ed.; *DSM-5*; American Psychiatric Association, 2013). Activity in these two areas could tentatively be seen as a biomarker for pragmatic competence, although our results at the same time show that there is no one such thing as pragmatic competence (e.g., as correlations between pragmatic tasks are not very high; see further discussion on the relation between different pragmatic measures in the supplemental discussion). If high skill participants standardly recruit these two areas to a larger degree when establishing communicative meaning, the consequence might be increased connectivity with other areas involved in pragmatic processing for these participants or enhanced (e.g., more relevant or more detailed) representation of speech acts for the HS participants in these areas.

### Limitations

Regarding limitations of the current study, it should be noted that we can really only make conclusions regarding the exact core language, ToM, and cognitive control aspects we measure through the selected tasks. A different selection of tasks may have manifested itself differently. While we partly base our statements about segregation on nonsignificant behavioral and brain-behavioral correlations, we want to emphasize that we do not base our conclusions on these alone. We also use converging evidence from locational information on the overlap between the clusters in the interaction analysis and the functional networks we study. The neurocognitive separation from the language network is clearer than the segregation from domain-general ToM, although we think we have also demonstrated some level of segregation with aspects of ToM (e.g., complex emotion recognition). From a skeptical point of view, if the individual differences in the ISA experiment were interpreted as reflecting domain-general processes rather than pragmatic processes, our data (e.g., the left lateral parietal and IPS activation) might be considered to point to attentional (e.g., feature selection) rather than cognitive control processes (e.g., including maintenance, manipulation, and inhibition). An additional limitation of the study is that we do not have the behavioral data to test whether the HS group would perform better or faster, e.g., as a consequence of using higher order areas when processing the dialogues. Finally, we would like to clarify that while we suggest that the left lateral parietal and precuneus findings here should be understood as indexes of making communicative inferences, we do not suggest that these are the only areas involved in communicative inferences (see supplemental discussion S3.4).

### Conclusion

We interpret our data as suggesting that pragmatics should be reduced to neither (1) an extension of core language processes in special circumstances nor (2) domain-general ToM/complex emotion processing or its combination with core language processes, even though we cannot rule out there being partly shared processing. We suggest that the highly replicable contrast of passive observation of indirect vs. direct speech could reflect low-level pragmatic processing, whereas areas in the lateral parietal lobe and dorsal precuneus, that are more active in this contrast for high communicative skill participants, could reflect more high-level pragmatic processing. In addition, we suggest that individual differences in the ISA experiment should be interpreted as reflecting the establishment of communicative inference—a pragmatic process that our evidence indicates is related to other pragmatic processes that we measured. While the data show some interrelations between different pragmatic tasks, additional research would be needed to exclude the possibility that the individual differences in pragmatic processing in the ISA experiment are partially related to differences in some domain-general aspect of cognition that we did not measure. In conclusion, we suggest that contextualized (and multimodal) communication requires interrelated and partly uniquely communicative skill sets that cannot simply be reduced to other seemingly related cognitive skills, such as core language skills, ToM skills, or cognitive control.

## ACKNOWLEDGMENTS

This research was funded by the Promobilia Foundation, Riksbanken Jubileumsfond, the Swedish Collegium of Advances Studies, and the Magnus Bergvall Foundation. Data acquisition was supported by a grant via the Stockholm University Brain Imaging Centre (SU FV-5.1.2-1035-15). We would also like to thank Daniel Pedersen for contributing to the translation of the AR task to a Swedish audience and Salomi Asaridou for important feedback on the design and manuscript.

## FUNDING INFORMATION

Katarina Bendtz, Stiftelsen Promobilia. Julia Uddén, Riksbankens Jubileumsfond (http://dx.doi.org/10.13039/501100004472). Julia Uddén, Swedish Collegium of Advanced Studies. Julia Uddén, Magnus Bergvalls Stiftelse (http://dx.doi.org/10.13039/501100006285). Julia Uddén, data acquisition supported by a grant to the Stockholm University Brain Imaging Centre, Award ID: SU FV-5.1.2-1035-15.

## AUTHOR CONTRIBUTIONS

**Katarina Bendtz**: Data curation; Formal analysis: Lead; Funding acquisition: Equal; Investigation: Lead; Methodology: Equal; Project administration: Equal; Software; Visualization; Writing – original draft: Equal; Writing – review and editing: Equal. **Sarah Ericsson**: Methodology: Supporting; Writing – review and editing: Supporting. **Josephine Schneider**: Investigation: Supporting; Writing – review and editing: Supporting. **Julia Borg**: Investigation: Supporting. **Jana Bašnáková**: Writing – review and editing: Supporting. **Julia Uddén**: Conceptualization; Formal analysis; Funding acquisition: Equal; Methodology: Equal; Project administration: Equal; Resources; Supervision; Writing – original draft: Equal; Writing – review and editing: Equal.

## TECHNICAL TERMS

Theory of speech acts: A perspective on utterances as conveying not only literal information but also an action, such as a request.
Pragmatics: Communication beyond what is given in the literal message.
Indirect speech act: A speech act conveying information that is not explicitly given literally, but is nonetheless expressed. “It would be nice with some coffee” is an example of an indirect request.
Audience design: The adjustment of utterances to suit the needs of interlocutors.
