# Supplementary material for: Individual Differences in Indirect Speech Act Processing Found Outside the Language Network
Source: Neurobiol Lang (Camb). 2022 Apr 13;3(2):287–317. doi: 10.1162/nol_a_00066 (PMC10158615; doi:10.1162/nol_a_00066)
Supplement: Supplementary file 1 [file nol-3-2-287-s001.pdf]

S1 Supplementary material

S1.1 Participants

Two participants reported dyslexia, one reported a history of dyslexia, and two participants reported previous logopedic therapy due to lisping or problems pronouncing “r”. These were included, as they were considered to be mild cases (there was no apparent effect on e.g. reading times).

**Number of participants (N) in each of the tasks**	
**Task**	**N**
PC-RR	199
AD	195
RMET	199
OSpan	194
AR	199
ISA	57
LDT	56
vSweSAT	57

Table S1. Number of participants (N) in each of the experiments included in the study.

S1.1.1 Behavioral experiment

For the Behavioral experiment, we invited a random sample of residents in the Stockholm area aged 18 – 35 whose phone numbers were registered in a publicly available database. Behavioral measurements were performed on 201 participants. Native knowledge of Swedish was an inclusion criterion which turned out not to be met by two participants, who were thus excluded. The final sample thus consisted of 199 participants. The data for the AD test was accidently deleted for an additional participant. Four participants had missing data in AD and could thus not be selected for the fMRI-experiment. Five subjects were unable to finish the OSpan task because they exceeded the time limit.

S1.1.2 fMRI-experiment

Using a two-sample t-tests, there was no significant difference neither between the durations ($p = 0.9$ (contexts), $p = 0.07$ (questions) and $p = 0.18$ (answer)) nor between the number of words ($p>0.58$) in neither the context, question nor answer (literal answer is the same across conditions). The reason as to why the questions are slightly longer in the Indirect trials is due to the fact that these often included more hesitation. Reducing this difference would have resulted in compromising with the ecological validity of the recordings. Crucially, we modeled the answers separately and the indirectness effect refers to the difference between direct and indirect conditions in the answers alone.

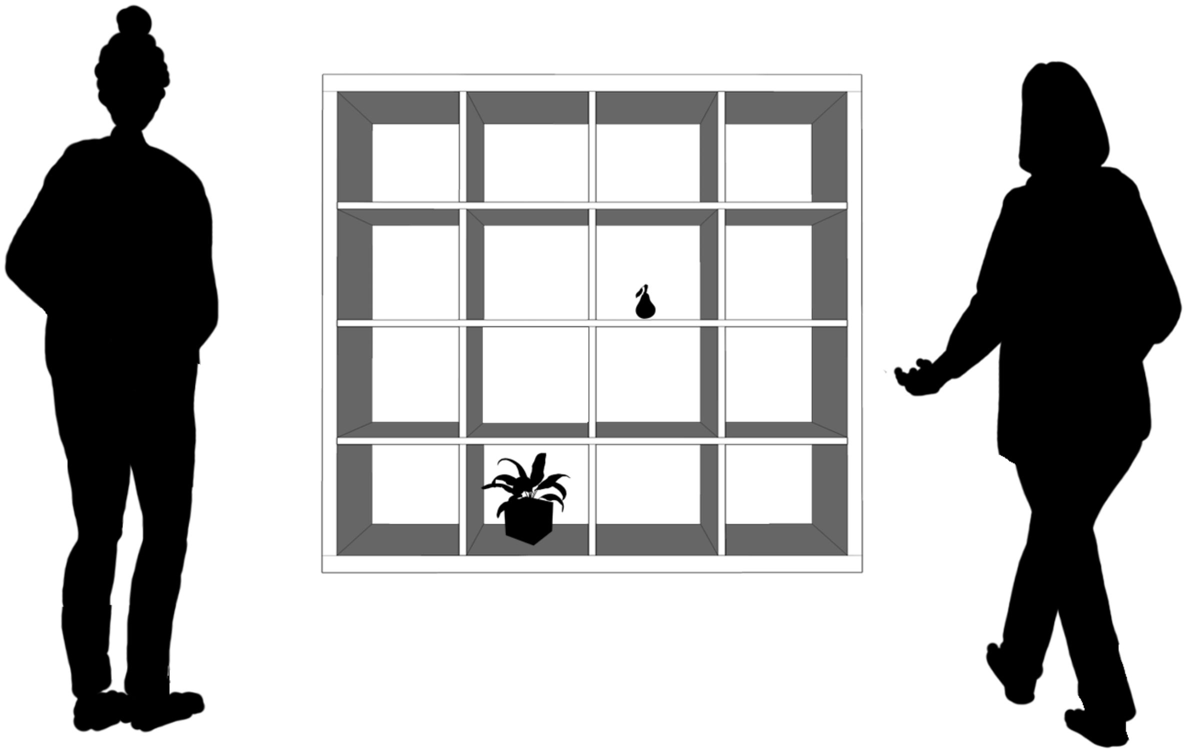

Figure S1. Cartoon showing the bookshelf from the AD test and two example objects along with a speaker (left) and an addressee (right). The drawing was shown to the participants before starting the experiment and the objects are therefore not taken from the real experiment.

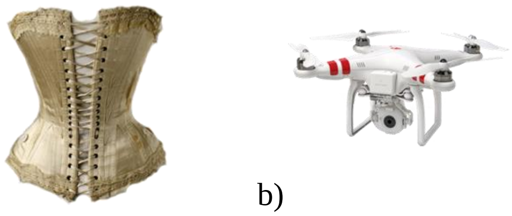

a) b)

Figure S2. Examples of objects appearing in the AD test.

S1.2 Pragmatic tests

S1.2.1 Production: Advanced Audience Design (AD)

Paraphrasing the object in a control trial resulted in -1 point to account for the possibility that the participant used paraphrasing as an overall strategy.

All participants did the same 20 trials (10 test trials, 10 control trials). The order of the trials was pseudo randomized resulting in four different versions. There were three addressees: a 6 year old child, a 91 year old person and a 31 year old person who moved to country Sweden (where the study was performed) only 6 months ago. The bookshelf (see Figure S1) was shown to the participant in 10 s during which the participant could describe the object (see Figure S2).

S.1.2.2. Comprehension: Pretest of Stimuli for Prosodic Comprehension of Request for Response (PC-RR)

The stimuli in the Prosodic Comprehension of Request for Response (PC-RR) task were tested in a pretest study, on a sample of 10 participants (no participant overlap with the main study). The purpose was to ensure that the stimuli were suitable for distinguishing individual differences in pragmatic ability. Hence, the pretest entailed presenting participants with the intended stimulus items and then removing items that were either too hard or too easy for participants to assess correctly. The pretest also showed that participants could distinguish between requests for response and neutral statements correctly on average 83% of the time, which corresponds well with the average performance in the main study.

S1.3 Cognitive control - Operation Span (OSpan)

The participants completed five blocks (order randomized), where one block corresponds to one letter sequence of 3, 4, 5, 6, or 7 letters. In an identical procedure to the "Partial Unit Scoring" (Turner & Engle, 1989) in Ryskin (2015), the score was determined by averaging the correctness ratio, *n_c/n_tot* over blocks (where *n_c* is the number of letters recalled correctly and *n_tot* is the total number of letters presented in that block). The other scoring methods found in the literature were rejected because they did not distinguish between trials where none of the letters on one hand, and where a fraction of the letters on the other hand, were correctly recalled. We judged this information important when studying individual variation.

S1.4 Behavioral tests of language ability

The participants completed the vocabulary test from the Vocabulary Scholastic Aptitude Vocabulary Test (as of fall 2016).

S1.4.1 Vocabulary as representative of "core language skills"

The most important and commonly measured language skills are probably receptive vocabulary, receptive grammar and expressive language ability (see further Wilson & Bishop (2019)). While we measure vocabulary, we did not include a test of grammar. There are tests of syntactic ability for children and aphasic patients, but there are yet no short, validated and reliable tests measuring adult variability in syntax in the normal population. However, we note that Wilson & Bishop (2019) devised a new grammatical decision test that did show a moderate correlation with vocabulary skills, but no correlation with pragmatic processing. This supports the view that our

vocabulary measures can be considered a representative skill for a set of inter-related core language skills.

S1.4.2 Author Recognition Test

220 names, whereof half real authors and half foil names, were presented to the participant in two separate sets. The order of the names was alphabetical, rendering different orders for each participant. The participant had been instructed to select those names that she knew or thought to be a real author, and that half of the names were foil names. The participants were further informed about the scoring procedure: the score is calculated by subtracting the incorrectly selected foils from the correctly selected real authors.

S1.4.2.1 Translating the author names to a Swedish audience

In the Moore study, the author list had been based on the original ART (Stanovich & West, 1989), and then revised so that it “reflected a mix of classic and more recently popular authors”. They further report “replacing authors who had extremely high or extremely low identification rates, so as to settle on a list of authors of generally moderate familiarity to our sample”. An Item Response Theory analysis revealed that the test was most informative for high abilities, (see Figure 2 in Moore (2015)) and the authors therefore recommend increasing the proportion of easy to moderate authors.

Moreover, Moore (2015) reported a roughly linear relation between the difficulty level b (for b < 10), of an author, and the log 10 of the frequency of the author name as it appears in the Corpus of Contemporary American English (COCA), which contains 450 million words drawn from sources published from 1990 to 2012, including fiction, magazines, newspapers, and academic journals (Davies, 2008). Inspection of Figure 4 in Moore however leads us to the conclusion that the linearity is not robust for b > 5, which we will assume in our analysis to be conservative.

We had two aims with the translation of the ART as given in Moore (2015). Firstly, we wanted to modify the difficulty level of the test so as to better test author knowledge in the whole ability range. Secondly, we wanted to adjust the author list to a Swedish audience.

In order to do this, we first determined the desired distribution of authors in terms of difficulty and then estimated how the frequency in a Swedish corpus corresponded to these difficulty levels. We further performed an Item Response Theory analysis on a preliminary version of SART where in total 35 items had been added and then chose the 65 items that optimized the sensitivity of the test. The authors used in all of the tests were assembled by the Swedish literature studies researcher Dr. Daniel Pedersen, ensuring a considerate diversity in the author names.

The sensitivity of the test is in this case defined as the precision of a test in the estimation of a certain ability. Within Item Response Theory this is given by the Test Information Function (TIF). In Moore (2015) this was peaked at an ability level way above average at around theta= 2.5, and our aim was to have an ITF symmetrically distributed around average ability. (Assuming here that b=0 is average ability.) To obtain the TIF of a test however, one would have to know the

discrimination parameter, a, and the difficulty parameter, b, for each item (to be able to estimate its Item Response Function). These parameters need to be estimated from data for each item, and we could thus not a priori determine a perfect set of items for our test. We can however still make a best guess using the information we have.

For a given a, the Item Information Function of each item is maximum at the ability level that corresponds exactly to the difficulty level of the item, by definition this is the ability that gives 50% probability of knowing the author. In other words, for this ability the item has the highest precision. It is then fair to assume that, since the Test Information Function is the sum of all Item Information Functions, a flat distribution in terms b is the best guess in order to obtain our desired Test Information Function. Using the linear proportionality between b and log10(frequency), we can a priori make sure that our items are largely homogeneously distributed in terms of b, keeping in mind that the proportionality does not hold for the most difficult authors.

In accordance with the above reasoning, an analysis of the Moore data gives that the distribution of b is bell-shaped rather than flat, skewed in favour of more difficult items and centered with a peak at about b = 2 and an average of b = 3.1, which could then possibly partly explain the skewness of the TIF.

Mediearkivet (Retriever (1996-)) was chosen as the Swedish correspondence to COCA, a fulltext archive of articles from newspapers from Sweden, trade journals, and news agencies. We used the frequencies calculated over a 5 year period (2013-08-28 - 2018-08-28). We defined five

categories, Ci, or author frequencies, as by $min_i < \log(freq_{Ci}) < max_i$, $1 < i < 5$ and selected an equal amount of authors, 20, in each category. This way we would obtain a roughly flat distribution of authors in terms of log(freq). The min_i and max_i were determined from data of a preliminary version of SAR (SAR_prel_1) which was administered with twelve participants. The aim of the preliminary study was mainly to get an estimate of how selection rate corresponded to frequency, so that we could draw conclusions about which frequency roughly corresponded to ceiling and which frequencies corresponded to authors in the difficulty range where the proportionality between log frequency and difficulty does no longer hold. The selection frequency is our best measure of difficulty level b of an item in absence of a full Item Response analysis. (An analysis of the Moore data revealed that the relationship between selection rate and item difficulty is linear in the same range as item difficulty is proportional to log10 frequency.)

The max_5 was defined as the average frequency of the authors with 100% selection rate (N=5) plus the standard deviation (to be conservative) (max_5 = (log10 (frequency_100_avg + sd_frequency_100). The linearity between log10 frequency and item difficulty stops being linear at about a selection rate of 10%. For lower selection rates, the difficulty level grows exponentially. We therefore decided to set the min_1 log(average frequency of the authors with < 8% selection rate <12% (N=5) of the SAR_prel_1). An additional category, C_0, was formed for the authors with a frequency < min_1, and the number of authors in this category was set to 10 to be conservative.

This preliminary SAR, SAR_prel_2, was tested on a sample of 200 participants. The data was then analysed with Response Theory analysis to select the 65 authors out of the total 110 which optimized the TIF and had the best individual discrimination parameters a.

The foil author names were chosen from two lists of names published online to advocate for a political cause. We made sure not to be authors or considerably known for something else. We also matched the foils to the real author names in terms of female sounding names and foreign sounding names,

No authors with names that were too common to ensure that the frequency in MA was not polluted significantly by non-relevant hits, were excluded. No authors which are mainly known for their work in some other profession were included.

S1.5. Analysis of behavioral data: RIN-transformation

A simulation study on non-normal data (Bishara & Hittner, 2012) evaluated different approaches to test the significance of a correlation given non-normal data. This study shows RIN-transformation to be the best choice for large sample sizes (N>160) like we have, considering both power and type 1 error for small to moderate correlation coefficients, (rho = 0.1 - rho = 0.5) which is what we expected. In contrast to the significance estimation, it remains an open question as to whether the correlation coefficient itself can be reliably estimated using any of the existing methods given non-normal data, and therefore we will not draw conclusions from the value of our

estimated coefficients. Along the same lines, we have refrained from any additional analysis on the RIN transformed data like partial correlations, as to our knowledge, no studies to ensure the reliability of such analyses using non-normal data exist.

S1.6 fMRI paradigm

The dialogs were recorded by ten males and seven females, all native language Swedish speakers. The contexts and compliance questions were all recorded by the same female speaker.

The recordings were manually edited in Logic X Pro: contexts were merged with dialogs, silences before (after) the dialogs were removed and the volume was matched over trials. Breathing sounds before or after the dialogs were included only if they were judged bearing communicative meaning.

S1.7 fMRI procedure

Participants received scripted oral instructions about the ISA experiment. We informed the participants that this was the main task and that occasional compliance questions only served as a means of checking the participant’s attention. An example of a direct dialog and a compliance question was also read to them. The first 9 minutes consisted of resting state measurements (results are not reported here). The ISA followed, preceded by a short recap instruction and an audio calibration. The runs were 10-12 min each, with a short break in between, the participants remaining in the scanner. A final structural scan of 4 min was recorded. Throughout the whole experiment, a cross-hair was present on the screen, with the exception of the duration of compliance question presentations. At those occasions, the text “Answer the question: NO or YES” was presented. The participants could choose to close their eyes or not but were instructed to focus

on the cross hair if they kept their eyes open. The trials as well as the compliance questions were presented in stereo via headphones. Some latencies due to a technical issue with the stimulus presentation was corrected for post hoc.

S1.8 Other measurements and analyses

We also measured a third pragmatic task (essentially a production version of the PC-RR task we introduce above), but as this the task did not meet our standards for inter-rater scoring reliability, we do not report it further.

Finally, we will here report an additional analytic approach for completeness. In addition to the whole-brain analysis reported in this paper, we performed four analyses where we constrained the tests to specific sets of cortical regions. The first three sets were determined using functional activation maps from previous research with almost identical paradigms (Asaridou et al., 2019; Bašnáková et al., 2014). Activated regions were divided into three functional classes: pragmatic, pragmatic/language and language activations, respectively, based on previous literature. The fourth brain region was defined using the ToM uniformity mask from neurosynth (Yarkoni et al., 2011). As this analysis did not yield significant results, this analysis in not reported in further detail.

S2 Supplemental Results

For an axial section view of the parietal and precuneus clusters, see Figure S3(B). The brain-behavioral correlations corresponding to the precuneus cluster are shown in Figure S4.

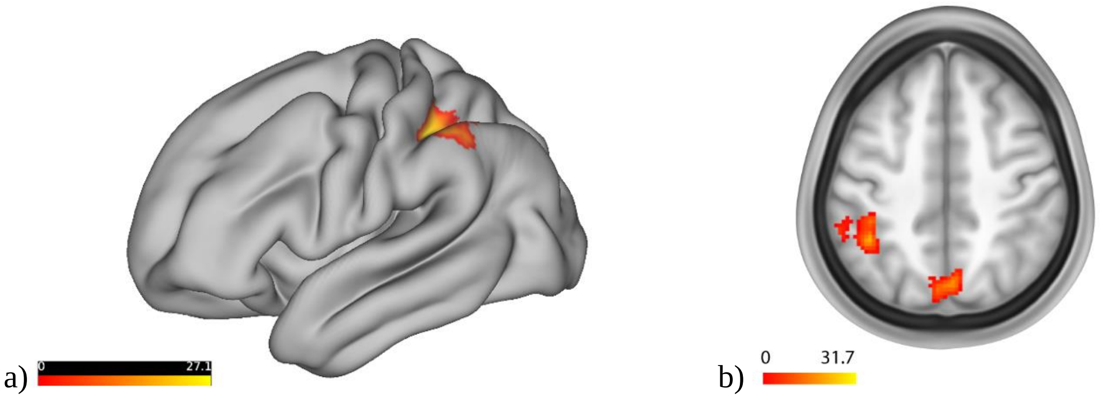

Figure S3. Brain activation of the indirect vs direct contrast, interaction between groups. The figure shows F-values for clusters with a cluster-forming threshold of $p_{uncorrected}=0.001$ (no extent-level threshold, $k = 0$). As a multiple comparison correction method, we used Family Wise Error (FWE) correction at the cluster- and peak level. We only report clusters and voxels with a $p_{FWE}<0.05$. A) The parietal cluster is projected onto a pial surface. B) An axial section at $z = 47.5$ showing both the parietal and precuneus clusters.

	Pragmatics tests		CCF	ToM	Language tests		
	AD<br>N = 194	PC-RR<br>N = 198	OSpan<br>N = 198	RMET<br>N = 193	AR<br>N = 198	vSweSAT<br>N = 60	LDT<br>N = 58
**AD**	-	p = 0.077<br>(ρ = 0.13)	p = 0.14<br>(ρ = 0.11)	p = 0.92<br>(ρ = $-7.6\cdot10^{-3}$)	p = 0.010<br>(ρ = 0.18)	p = 0.012<br>(ρ = 0.32)	p = 0.79<br>(ρ = 0.035)
**PC-RR**		-	p = $2.9\cdot10^{-3}$<br>(ρ = 0.21)	p = 0.43<br>(ρ = 0.057)	p = $8.2\cdot10^{-3}$<br>(ρ = 0.19)	p = $4.6\cdot10^{-4}$<br>(ρ = 0.44)	p = 0.19<br>(ρ = 0.18)
**OSpan**			-	p = 0.92<br>(ρ = $7.2\cdot10^{-3}$)	p = 0.042<br>(ρ = 0.15)	p = $5.6\cdot10^{-4}$<br>(ρ = 0.43)	p = 0.17<br>(ρ = 0.18)
**RMET**				-	p = 0.74<br>(ρ = -0.024)	p = 0.24<br>(ρ = -0.16)	p = 0.024<br>(ρ = 0.30)
**AR**					-	p = $9.2\cdot10^{-6}$<br>(ρ = 0.54)	p = 0.72<br>(ρ = 0.049)
**vSwe SAT**						-	p = 0.47<br>(ρ = 0.098)

Table S2. Two-tailed Pearson correlation tests on RIN-transformed data for the behavioral tests. Correlation values are shown in parenthesis.

S3 Supplemental discussion

S.3.1 The indirect vs direct effect, in both groups - continuation

It can be noted that the HS and LS groups both activated regions that are covered by the neurosynth 'language comprehension' association map in three regions: (L1, L for Language) bilateral IFG, (L2) bilateral anterior temporal lobe and (L3) right mid MTG/STS. In addition, the LS group showed left posterior MTG/STS activity (a result which could be suggestive of a more "literal" processing style), but there was no interaction between groups in this region. In addition, the HS and LS groups both activated two regions covered by the neurosynth 'ToM' association map: (T1, T for ToM) medial SFG/dmPFC, (T2) bilateral TPJ.). The HS group activated a larger portion of cortices, ventrally and dorsally of the left TPJ. This group also showed significant activity (absent in the LS group) in the precuneus, a pattern which partly resulted in a significant group interaction. The more dorsal parts that interacted significantly (z ~55) were outside the neurosynth 'ToM' association map precuneus cluster, while the part that did not interact (for instance [-4, -68, 34], see Table 7), were closer to the ventral (z ~ 35) cluster in the neurosynth map.

It can be noted that table 8 in the main manuscript shows an overlap between the superior parietal cluster in the interaction and one of the 'cognitive control' neurosynth maps. While some of the areas (IFG, medial SFG/dmPFC, TPJ and the ventral z ~ 35 precuneus cluster) reported in the HS and LS groups, separately or in the overlap between groups, bordered the 'Cognitive Control' neurosynth map, there was no substantial overlap.

Due to these activation differences between groups, the reader might ask whether we believe that the participants would actually show differences in behavior in the fMRI ISA experiment, if we had probed their performance in this task in more detail. We believe it is likely that there would

be behavioral differences across groups in the ISA experiment, at least in the timing and processing cost needed to draw a correct inference, for the following reasons: (1) pragmatic skills are interrelated, as shown in the current paper and by others (Wilson & Bishop, 2019). (2) The HS group show higher activation in high order areas. While we do not have the behavioral data to test this, we suggest it is more probable that the HS group would perform better or faster, e.g. as a consequence of using higher order areas when processing the dialogs. The HS group might have recruited additional regions, e.g. the lateral parietal and dorsal precuneus cluster we find, on top of a *core, or lower-level,* pragmatics network.

S3.2 Inferior and superior parietal cortex

Inspection of Figure 2 in the main result section shows that for the individual signal intensities in the indirect vs direct contrast in the cluster in the superior parietal cortex, the HS group participants generally have positive values and LS group participants have negative values (although there is a unimodal distribution with a mean around or slightly above 0). This could be interpreted as a consequence of different mechanisms used across participants, where both possible mechanisms involve this area but in different ways.

For the inferior parietal cortex, although we think the interpretation we give in the main manuscript is much more likely, we cannot exclude the possibility that this part of the cluster is actually driven by some kind of non-pragmatic conceptual activity that would still differ across indirect and direct conditions and correlate with pragmatic skill across individuals.

As we have noted in the main manuscript, the right hemisphere area corresponding to the left lateralized cluster we report does not show a significantly different pattern (compared to its left counterpart). Thus, the absence of a corresponding right hemispheric effect is a result of the

activations being slightly below the statistical threshold, rather than representing more substantial differences across hemispheres. This is a methodological perspective that often lacking in the literature, and we therefore take available suggestions (Ciaramidaro et al., 2007; Enrici et al., 2019) on functional differences for the left and right lateral parietal area in intention processing lightly.

S3.3 Behavioral results: Relation between pragmatic production and comprehension

From the psycholinguistic perspective we provide evidence for a relation between different pragmatic processes, e.g. between pragmatic production and comprehension. As we used a broad approach to measure individual variation in pragmatic ability, we included both comprehension and production tasks. As we are not aware of previous studies on the relation between individual variance in comprehension and production, we tested whether the seemingly varied pragmatic skills in our battery would be partially inter-related, when combining neural and behavioral measures. For instance, it was far from clear that we should expect individual proficiency to be related across production and comprehension. There is a branch of psycholinguistics that considers production and comprehension as highly inter-related, across the board. Garrod and Pickering (2015) suggest that listeners use their production system to predict everything from phonetics to speaker intentions. Our results of significant correlations from a comprehension test (the PC-RR task) with the audience design production task is thus an empirical extension of the suggestion of a general close relation of production and comprehension (Garrod & Pickering, 2015) for pragmatic processes. This indicates shared aspects of neurocognitive characteristics of comprehension and production of communicative intent (e.g. shared segregation from the core language system). Predictions from pragmatic production processes could be used for increased proficiency in pragmatic comprehension (Pickering & Garrod, 2007). Some set of core pragmatic representations could be shared and used across production and comprehension, a possibility that

should be further explored.

S3.4 Clarification: our findings should not be interpreted as all areas involved in communicative inferences

While our interpretation of the main pattern of results is that the process of establishing communicative meaning cannot be reduced to core language processes, we want to stress that the regions we report should not be taken as all areas involved in communicative inferences. Several additional areas, e.g. the ones reported in the whole group averages, likely contribute in different ways as well, with more or less specificity. This most likely includes the regions from the indirect vs direct contrast observed in both groups. Thus, there is likely to be *some* overlap of the neural infrastructure subserving core language and pragmatic processing. Our data however brings evidence for the position that such an overlap importantly cannot be the whole story. Using the individual difference approach, we contribute by pinpointing two clusters: the precuneus and the parietal cortex.

References

Asaridou, S. S., Demir-Lira, Ö. E., Uddén, J., Goldin-Meadow, S., & Small, S. L. (2019). Pragmatic Language Processing in the Adolescent Brain. *bioRxiv*, 871343. https://doi.org/10.1101/871343

Bašnáková, J., Weber, K., Petersson, K. M., van Berkum, J., & Hagoort, P. (2014). Beyond the language given: the neural correlates of inferring speaker meaning. *Cereb Cortex*, *24*(10), 2572-2578. https://doi.org/10.1093/cercor/bht112

Ciaramidaro, A., Adenzato, M., Enrici, I., Erk, S., Pia, L., Bara, B. G., & Walter, H. (2007). The intentional network: How the brain reads varieties of intentions. *Neuropsychologia*, *45*(13), 3105-3113. https://doi.org/https://doi.org/10.1016/j.neuropsychologia.2007.05.011

Enrici, I., Bara, B. G., & Adenzato, M. (2019). Theory of Mind, pragmatics and the brain: Converging evidence for the role of intention processing as a core feature of human communication. *Pragmatics & Cognition*, *26*(1), 5-38. https://doi.org/https://doi.org/10.1075/pc.19010.enr

Garrod, S., & Pickering, M. J. (2015). The use of content and timing to predict turn transitions [Hypothesis and Theory]. *Frontiers in Psychology*, *6*(751). https://doi.org/10.3389/fpsyg.2015.00751

Pickering, M. J., & Garrod, S. (2007). Do people use language production to make predictions during comprehension? *Trends Cogn Sci*, *11*(3), 105-110. https://doi.org/10.1016/j.tics.2006.12.002

Ryskin, R. A., Benjamin, A. S., Tullis, J., & Brown-Schmidt, S. (2015). Perspective-taking in comprehension, production, and memory: An individual differences approach. *Journal of Experimental Psychology: General*, *144*(5), 898-915. https://doi.org/10.1037/xge0000093

Stanovich, K. E., & West, R. F. (1989). Exposure to print and orthographic processing. *Reading Research Quarterly*, *24*(4), 402-433. https://doi.org/10.2307/747605

Turner, M. L., & Engle, R. W. (1989). Is working memory capacity task dependent? *Journal of Memory and Language*, *28*(2), 127-154. https://doi.org/https://doi.org/10.1016/0749-596X(89)90040-5

Yarkoni, T., Poldrack, R. A., Nichols, T. E., Van Essen, D. C., & Wager, T. D. (2011). Large-scale automated synthesis of human functional neuroimaging data. *Nature methods*, *8*(8), 665-670. https://doi.org/10.1038/nmeth.1635
